# Intellectual Disability and Potassium Channelopathies: A Systematic Review

**DOI:** 10.3389/fgene.2020.00614

**Published:** 2020-06-23

**Authors:** Miriam Kessi, Baiyu Chen, Jing Peng, Yulin Tang, Eleonore Olatoutou, Fang He, Lifen Yang, Fei Yin

**Affiliations:** ^1^Department of Pediatrics, Xiangya Hospital, Central South University, Changsha, China; ^2^Hunan Intellectual and Developmental Disabilities Research Center, Changsha, China; ^3^Kilimanjaro Christian Medical University College, Moshi, Tanzania; ^4^Mawenzi Regional Referral Hospital, Moshi, Tanzania

**Keywords:** intellectual disability, mental retardation, global developmental delay, potassium, channelopathies

## Abstract

Intellectual disability (ID) manifests prior to adulthood as severe limitations to intellectual function and adaptive behavior. The role of potassium channelopathies in ID is poorly understood. Therefore, we aimed to evaluate the relationship between ID and potassium channelopathies. We hypothesized that potassium channelopathies are strongly associated with ID initiation, and that both gain- and loss-of-function mutations lead to ID. This systematic review explores the burden of potassium channelopathies, possible mechanisms, advancements using animal models, therapies, and existing gaps. The literature search encompassed both PubMed and Embase up to October 2019. A total of 75 articles describing 338 cases were included in this review. Nineteen channelopathies were identified, affecting the following genes: *KCNMA1, KCNN3, KCNT1, KCNT2, KCNJ10, KCNJ6, KCNJ11, KCNA2, KCNA4, KCND3, KCNH1, KCNQ2, KCNAB1, KCNQ3, KCNQ5, KCNC1, KCNB1, KCNC3*, and *KCTD3*. Twelve of these genes presented both gain- and loss-of-function properties, three displayed gain-of-function only, three exhibited loss-of-function only, and one had unknown function. How gain- and loss-of-function mutations can both lead to ID remains largely unknown. We identified only a few animal studies that focused on the mechanisms of ID in relation to potassium channelopathies and some of the few available therapeutic options (channel openers or blockers) appear to offer limited efficacy. In conclusion, potassium channelopathies contribute to the initiation of ID in several instances and this review provides a comprehensive overview of which molecular players are involved in some of the most prominent disease phenotypes.

## Introduction

Once termed mental retardation, intellectual disability (ID) manifests prior to adulthood in the form of severe limitations to intellectual function and adaptive behavior (van Bokhoven, 2011). Potassium channels have diverse gating properties and wide-ranging expression profiles, which allows them to regulate cellular excitability during growth in numerous ways (Niday and Tzingounis, 2018) and moderate the repolarization rate of action potentials and membrane resistance, resting membrane potential, and spike frequency (Storm, 1990; Jan and Jan, 2012; Kole and Stuart, 2012; Niday and Tzingounis, 2018). Some are expressed in oligodendrocyte progenitors (Schmidt et al., 1999) and dendrites, where they have significant influence on learning and memory (Vacher et al., 2008). Potassium channelopathies are associated with multiple neurological disorders related to ID and epilepsy. The burden of potassium channelopathies in ID is unknown. Gain-of-function mutations may lead to ID, whereas loss-of-function mutations leans toward epilepsy onset. However, this is not necessarily the case as gain-of-function mutations can also result in severe epilepsy (Niday and Tzingounis, 2018).

We hypothesized that potassium channelopathies played an important role in ID occurrence, with both gain- and loss-of-function mutations leading to ID. To this end, we have compiled a list of all potassium channel gene mutations previously reported to associate with ID. The list is complemented by current knowledge regarding possible mechanisms (gain- or loss-of-function), advancements in animal models, therapies, and existing gaps. This review aims to facilitate future studies on the mechanisms of ID and the identification of possible treatments. Unlike previous publications, it is also the first such study to systematically explore the relationship between potassium channelopathies and ID rather than epilepsy.

## Methods

### Literature Search and Selection

The review was conducted according to the Preferred Reporting Items for Systematic Reviews and Meta-Analyses statement. PubMed and Embase were thoroughly searched up to October 2019 (Moher et al., 2015). The following search strategies were utilized: ID and potassium channel, mental retardation and potassium channel, global developmental delay (GDD) and potassium channel (Supplementary Datasheet 1). The search strategies were devised in consultation with a librarian and were applied by two independent reviewers to select papers that met our review objectives.

The following types of studies were included: cohorts, case-controls, cross-sectionals, case series, and case reports. We selected papers that included patients with ID/GDD and potassium channel gene mutations. We excluded papers on patients with ID/GDD but who presented other types of channelopathies (sodium, calcium, and chloride) or other gene mutations. We further excluded studies that reported patients with potassium channelopathies without information related to the degree of ID/GDD or comment whether the patient had GDD. Finally, we excluded non-English papers, abstracts, reviews, patents, book chapters, and conference papers. Reference lists of published articles were hand-searched for secondary sources.

### Data Extraction

Two independent reviewers screened the titles and abstracts, and thereafter read the full texts of those that appeared to meet inclusion criteria. Accuracy of the extracted information was ensured through discussion and consensus. For articles that met inclusion criteria, we collected information related to potassium channel gene mutations, the associated phenotype on top of ID/GDD, severity of ID (mild, moderate, and severe), electrophysiological studies results (gain-of-function or loss-of-function), and the corresponding references. All identified pathology-associated genes were subjected to further analysis in OMIM and PubMed, ClinVar, and www.rikee.org (*KCNQ2* and *KCNQ3* mutations) databases to identify their function, expression, studies in animal models, available treatments, and possible mechanisms underlying ID/GDD.

## Results

The initial search yielded 458 articles. Following the elimination of duplicates and articles that lacked full texts and/or were non-English, 126 remained. All full texts were read and screened for eligibility; 52 did not meet inclusion criteria, whereas 74 did. Among the latter, however, we did not include an article by Burgess et al. (2019) since it lacked detailed information and it included previously reported cases. In addition, two articles regarding *KCNT1* and *KCNT2* published in 2020 were also included (Borlot et al., 2020; Mao et al., 2020) making a total number articles meeting the inclusive criteria to be 75 (Supplementary Flow Chart 1).

### Potassium Channelopathies Associated With GDD/ID and Their Functional Properties

**Figure 1:**
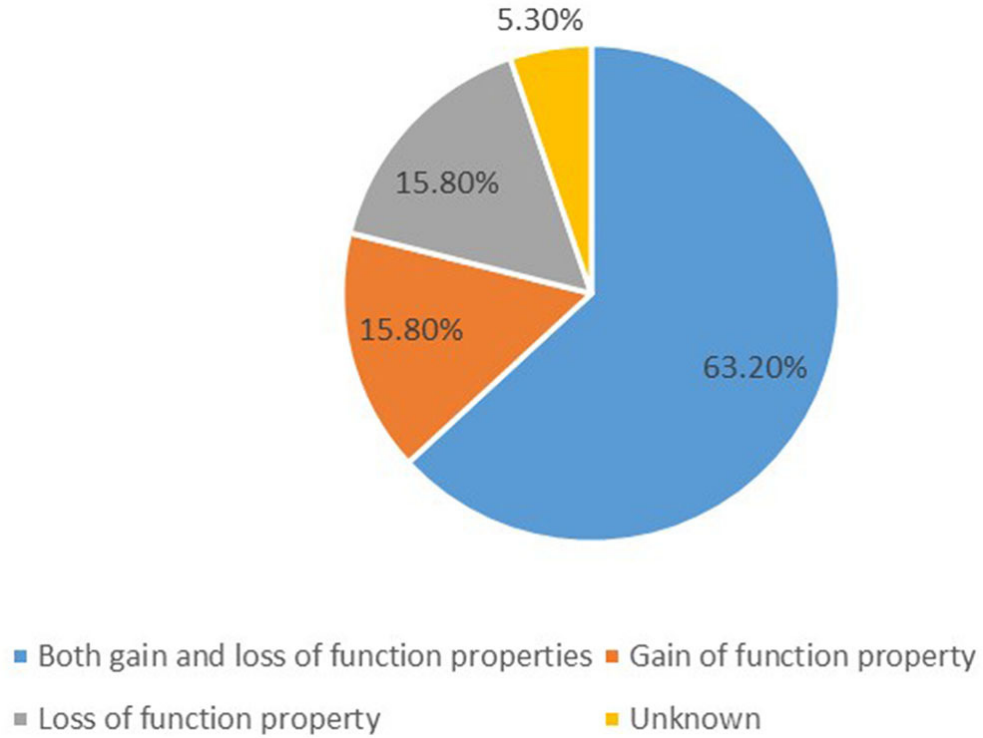
A summary of functional properties of the 19 identified potassium channels gene mutations.

We identified 19 potassium channel gene mutations that were associated with GDD/ID in 338 cases. These included: *KCNMA1, KCNN3, KCNT1, KCNT2, KCNB1, KCNJ10, KCNJ6, KCNJ11, KCNA2, KCNA4, KCND3, KCNH1, KCNQ2, KCNAB1, KCNQ3, KCNQ5, KCNC1, KCNC3*, and *KCTD3*. Those with both gain- and loss-of-function properties included *KCNMA1, KCNJ10, KCNJ6, KCNJ11, KCNT1, KCNT2, KCNA2, KCNA4, KCND3, KCNQ2, KCNQ3*, and *KCNQ5*; those characterized by only gain- of-function properties included *KCNN3, KCNH1*, and *KCNC3*; and those presenting only loss-of-function properties included *KCNC1, KCNB1*, and *KCTD3*, as summarized in Figure 1. The function of *KCNAB1* was unknown. These genes are associated with mild to profound ID/GDD, as detailed in Supplementary Table 1.

### Calcium-Dependent Potassium Channels

#### Big Potassium Channels

Big potassium (BK) channels are large-conductance Ca^2+^-activated and voltage-activated potassium channels present in various tissues (Contet et al., 2016). They are termed also Maxi-K, or Slo1. When open, BK channels cause a substantial efflux of K^+^ ions, leading to hyperpolarization of the cellular membrane (Contet et al., 2016). They can detect simultaneously increased intracellular Ca^2+^ levels and membrane depolarization (Contet et al., 2016). This property is useful in excitable cells, as it allows control of their activity via negative feedback regulation of Ca^2+^ influx through voltage-activated Ca^2+^ channels (Contet et al., 2016). BK channels localize to the plasma membrane of neurons in the central nervous system (CNS), where they modulate the shape, frequency, and propagation of action potentials, as well as the release of neurotransmitter from presynaptic terminals (Contet et al., 2016; Griguoli et al., 2016). BK channels are also present in the neurons' nuclear envelope, thereby controlling gene transcription and neuronal morphology (Li et al., 2014). Finally, their presence in astrocytes or vascular smooth muscle cells enables the regulation of blood flow in the brain, which can influence cerebral activity (Contet et al., 2016). Mutation of fragile X mental retardation protein (FMRP) is the leading cause of ID and autism spectrum disorder (Reymundo et al., 2014). FMRP regulates neurotransmitter release and the transmission of synaptic information by modulating the duration of action potentials via BK channels in hippocampal and cortical pyramidal cells (Deng et al., 2013; Deng and Klyachko, 2016; Ferron, 2016). Enhanced BK channel activity and reduced presynaptic glutamate release were shown to cause ID in *Crbn* knock-out (KO) mice, whereas BK channel blockers restored normal cognitive behavior (Choi et al., 2018). A BKCa channel opener molecule rescued hippocampal glutamate homeostasis as well as and cognitive impairments in *Fmr1* KO mice (Hebert et al., 2014). An increased BK channel activity has been shown to manifest as an amplified intrinsic excitability in individual neurons and consequent network synchronization; whereas BK channel blockers stabilized neuronal excitability in both human and mouse neurons and improved seizure susceptibility in an Angelman syndrome mouse model (Sun et al., 2019).

##### KCNMA1

*KCNMA1* (potassium calcium-activated channel subfamily M alpha 1) encodes the alpha-subunit of the BK channel (Bailey et al., 2019) and regulates synaptic neuronal excitability (Bailey et al., 2019). This gene is highly expressed in numerous parts of the human brain, including the cerebral cortex and hippocampus (Contet et al., 2016). Besides its role in innate immunity, the KCNMA1 channel controls neuronal excitability and neurotransmitter release, repolarization of the membrane, smooth muscle tone, and tuning of hair cells in the cochlea (Petersen and Maruyama, 1984; Murrow and Fuchs, 1990; Brayden and Nelson, 1992; Robitaille and Charlton, 1992; Wu et al., 1999). Both gain- and loss-of-function mutations of this gene have been reported to associate with ID/GDD, leading to a range of mild to severe phenotypes depending on the variant.

Liang et al. (2019) reported eight cases with loss-of-function mutations G375R, S351Y, N449fs, G356R, and I663V, which eliminated the BK current; as well as P805L and C413Y, which caused a reduction in the amplitude of the BK current and a shift to a positive potential for the activation curves. A case carrying the G375R variant presented with severe GDD, dysmorphic features, visceral malformations, bone dysplasia, and connective tissue abnormalities (Liang et al., 2019). The other cases presented with mild to severe ID, speech delay, ataxia, axial hypotonia, and cerebral atrophy (Liang et al., 2019). Similarly, Laumonnier et al. (2006) reported a patient with severe ID, epilepsy, and autism spectrum disorder, who carried the A138V substitution, indicating that the mechanism of this variant was haploinsufficiency.

Gain-of-function mutations, such as D434G and N995S, have been reported to associate with generalized epilepsy and paroxysmal dyskinesia (Du et al., 2005). Two cases that presented with GDD, epilepsy, severe cerebellar atrophy, and carried loss-of-function mutation (Y676Lfs*7) were reported (Tabarki et al., 2016). Two Chinese boys, who presented with GDD and paroxysmal non-kinesigenic dyskinesia, had either the E884K or N1053S variants, but again no functional study was carried out (Zhang Z. B. et al., 2015). Yesil et al. (2018) described a patient with a homozygous truncating mutation (R458^*^), who presented with severe ID, epilepsy, corticospinal-cerebellar tract atrophy, and paroxysmal dyskinesia. His phenotype was explained by both loss- and gain-of-function mechanisms. Thus, it seems that both gain- and loss-of-function mutations in *KCNMA1* are related to ID. Currently, there is no animal model for the *KCNMA1* gene.

#### Small Potassium Channels

Small potassium (SK) channels are important for learning and memory as they are expressed in the postsynaptic membrane of glutamatergic synapses (Adelman et al., 2012). There, they control synaptic transmission and stimulate synaptic plasticity (Adelman et al., 2012). It has been shown that an increase in SK channels activity impairs learning (Vick et al., 2010; Adelman et al., 2012); whereas SK channel blockers ameliorate learning and memory in animal models (Hammond et al., 2006; Lin et al., 2008).

##### KCNN3

*KCNN3* encodes one of three members of the small-conductance calcium-activated potassium channels (SK3 channels) (Sailer et al., 2002). SK3 channels are abundant in the hippocampus, where they regulate memory and learning (Sailer et al., 2002). They are voltage-independent and gated by submicromolar intracellular calcium levels (Sailer et al., 2002). They form large multiprotein complexes comprising of pore-forming channel subunits, constitutively bound calmodulin calcium sensor, protein kinase CK2, and protein phosphatase 2A (PP2A) (Xia et al., 1998; Bildl et al., 2004; Allen et al., 2007). Binding of calcium ions to calmodulin opens SK channels (Xia et al., 1998). CK2 and PP2A phosphorylate or dephosphorylate SK-bound calmodulin, thus further controlling calcium sensitivity of the channels (Bildl et al., 2004; Allen et al., 2007; Adelman et al., 2012). Bauer et al. (2019) reported three cases diagnosed with Zimmermann–Laband syndrome and carrying either one of the following *de novo* missense variants: S436C, K269E, and G350D. They presented with mild to moderate ID, epilepsy, facial dysmorphism, hypertrichosis, and gingival overgrowth (Bauer et al., 2019). Electrophysiological studies revealed gain-of-function as an underlying mechanism for this syndrome (Bauer et al., 2019). Gain-of-function mutations increases calcium sensitivity of SK3 channels resulting in higher open-state probability and conductance of KCNN3 mutant channels (Bauer et al., 2019). Targeted studies on *Kcnn3* knock-in mice will finally confirm the occurrence of ID through gain-of-function mutations in this gene.

### Sodium-Activated Potassium Channels

#### Slack Channels

##### KCNT1

*KCNT1* encodes the sodium-activated Slack channel, also known as Slo2.2, whose name derives from “sequence like a calcium-activated K^+^” (Kim and Kaczmarek, 2014). KCNT1 mRNA and protein are abundant in neurons across the brain, including in the frontal cortex and hippocampus (Bhattacharjee et al., 2002; Santi et al., 2006; Brown et al., 2008). A sodium-sensitive potassium current is elicited in various neuronal cell types following an inflow of Na^+^ through sodium channels or neurotransmitter receptors (Bhattacharjee and Kaczmarek, 2005). This current modulates the hyperpolarization that occurs following repetitive firing, as well as neuronal excitability and adaptation following repeated, high-frequency stimulation (Zhang et al., 2012; Ferron, 2016). Slack channels interact with downstream cytoplasmic signaling pathways, and dysregulation of this coupling could prompt the unusual association of ID and epilepsy (Fleming et al., 2016). They interact with FMRP (Brown et al., 2010), phosphatase and actin regulator 1 (Phactr1), and cytoplasmic FMR1-interacting protein 1 (Cyfip1) (Fleming et al., 2016). The FMRP/Slack interaction controls the probability that Slack channels would open (Brown et al., 2010; Zhang et al., 2012). The Phactr1/Slack interaction is entirely abolished in mutant Slack channels, which could explain the occurrence of severe ID and epilepsy (Fleming et al., 2016). Bausch et al. (2015, 2018) showed that this channel was important for cognitive flexibility and normal social behavior in mice.

Mutations in the *KCNT1* gene have been correlated with numerous phenotypes, the most common being epilepsy of infancy with migrating focal seizures, followed by autosomal dominant nocturnal frontal lobe epilepsy (ADNFLE) (Barcia et al., 2019). Other rare phenotypes include early infantile epileptic encephalopathy and severe dystonia (Gertler et al., 2019), severe ID and epilepsy (Alsaleem et al., 2019), myoclonic-atonic epilepsy and moderate ID (Burgess et al., 2019), Ohtahara syndrome (Martin et al., 2014), temporal lobe epilepsy, cerebellar ataxia and ID (Hansen et al., 2017), as well as leukoencephalopathy accompanied by severe epilepsy and severe ID (Vanderver et al., 2014; Evely et al., 2017; Burgess et al., 2019). Except for ADNFLE, a large proportion of cases with this channelopathy present with severe to profound ID (Supplementary Table 1). Electrophysiological studies have shown that gain-of-function (hyperactivation of slack channels) (Bearden et al., 2014; Martin et al., 2014; Rizzo et al., 2016; Kawasaki et al., 2017; Zhang et al., 2017; Dilena et al., 2018; McTague et al., 2018; Numis et al., 2018; Alsaleem et al., 2019; Barcia et al., 2019; Gertler et al., 2019; Borlot et al., 2020) and loss-of-function are the underlying mechanism contributing to the occurrence of ID (Evely et al., 2017).

Quinidine has been reported to rescue the gain-of-function effect of the K^+^ channel mutation *in vitro* (Milligan et al., 2014; Dilena et al., 2018). Nevertheless, its efficacy in clinical settings is variable, which casts doubts over gain-of-function being the underlying mechanism. Dilena et al. (2018) reported 2 cases with epilepsy of infancy with migrating focal seizures. They both carried gain of function mutations (either R950Q or E893K), and quinidine could reduce seizure burden by 90%, however, it could not normalize the milestones; both cases remained with severe GDD. In another study, four cases carrying gain of function mutations (either R1114W or A259D or M516V or R428Q) did not respond to quinidine (2 cases both *in vivo* and *in vitro*, and the remaining 2 *in vivo* only) (Numis et al., 2018). In addition, four cases in another study received quinidine, however, only one case showed >50% reduction of seizure frequency, and no comment was given about the status of milestones development (Yoshitomi et al., 2019). Likewise, one of the three cases responded to quinidine (>50% reduction in seizure frequency), as a result, the authors suggested that the response could be explained by the early age of treatment commencement (3 months) vs. (9 and 13 years, respectively) for non-responders (Abdelnour et al., 2018). Nevertheless, another report indicated that there was no benefit of quinidine on neither seizures nor milestones despite the age at treatment initiation (6 months) (Numis et al., 2018). Barcia et al. (2019) hypothesized that quinidine failed to improve developmental problems as it did not alter the non-conducting functions of *KCNT1*. *KCNT1* mutations can have a negative impact on the channel's gating properties, as well as its coupling to cytoplasmic signaling pathways as mentioned above. Thus, FMRP, Phactr1, and Cyfip1 are potential targets of novel therapeutic strategies, whereas future animal model studies will reveal the role of other proteins.

*Kcnt1* KO mice demonstrated eradicated sodium-sensitive potassium current and increased excitability in dorsal root ganglion neurons that resulted to more itching (Martinez-Espinosa et al., 2015). Despite the fact that authors did not perform cognitive tests, *Kcnt1* KO mice demonstrated normal ability to eat, mate, and function, as a result, authors speculated that the alteration of *Kcnt1* (gain-of-function) might lead to more deleterious consequences than the complete absence of *Kcnt1* (loss-of-function) (Martinez-Espinosa et al., 2015). This argument is supported by our review as majority of reported cases have gain-of-function mutations in contrast to one case with loss-of-function mutation. In another study, *Kcnt1* KO mice exhibited increased sensory neuron excitability that manifested as an exaggerated pain sensitivity (Lu et al., 2015). Altogether, the two studies (Lu et al., 2015; Martinez-Espinosa et al., 2015) suggest that loss-of-function mutations are likely to increase neuronal excitability while gain-of-function mutations are likely to reduce neuronal excitability. Nevertheless, both gain- and loss-of-function mutations have been reported to associate with ID as shown above. Therefore, how gain- and loss-of-function mutations lead to ID is yet to be explained by animal models. Cognitive tests can be carried out for *Kcnt1* KO mice, and there is a need of developing *Kcnt1* knock-in mice.

##### KCNT2

*KCNT2* (potassium sodium-activated channel subfamily T member 2), also known as Slo2.1 or Slick channel, is activated by intracellular Na+ and Cl- and inhibited by intracellular ATP (Bhattacharjee et al., 2003). It is expressed in different neurons in the CNS including the hippocampus and cortex (Bhattacharjee et al., 2005; Rizzi et al., 2016). KCNT1 and KCNT2 subunits can co-localize to form homo- or tetra-heteromeric channels (Chen et al., 2009). Like Slack channels, slick channels produce currents that modulate the hyperpolarization that occurs following repetitive firing, as well as neuronal excitability and adaptation following repeated, high-frequency stimulation (Zhang et al., 2012; Ferron, 2016).

*KCNT2* mutations have been reported to be associated with early onset epileptic encephalopathy (Gururaj et al., 2017), West syndrome advancing to Lennox–Gastaut syndrome (Ambrosino et al., 2018) and epilepsy of infancy with migrating focal seizures (Ambrosino et al., 2018; Mao et al., 2020). A total number of 5 cases have been reported so far, and all presented with severe ID (Supplementary Table 1). Three cases carried loss-of-function mutations (F240L, L48Qfs43^*^, and K564^*^) (Gururaj et al., 2017; Mao et al., 2020) while the remaining two carried gain-of-function mutations (R190P and R190H) (Ambrosino et al., 2018). One of the two cases reported by Ambrosino et al. (2018) was treated with quinidine which improved both seizures and milestones. Further studies with large sample size are essential to consolidate the benefits of quinidine in developmental progression. Despite the fact that *Kcnt1* KO mice demonstrated eradicated sodium-sensitive potassium current and increased excitability in dorsal root ganglion neurons that resulted to more itching, *Kcnt2* KO mice did not exhibit similar findings, and cognitive tests were not conducted (Martinez-Espinosa et al., 2015). *Kcnt2* KO mice exhibited normal sodium-sensitive potassium current and neuronal excitability. Consequently, there is a need of exploring the non-conducting functions of *KCNT2* since our review shows that loss-of-function mutations associate with ID. In addition, future studies can focus on understanding why *Kcnt2* KO mice did not have similar findings as *Kcnt1* KO mice. Finally, animal studies will unveil how gain- and loss-of-function mutations lead to ID.

### Inward Rectifier Potassium Channels

#### KCNJ6 (GIRK2)

*KCNJ6* (potassium inwardly rectifying channel subfamily J member 6) encodes the Kir3.2 GABAB receptor-coupled channel, a member of the G protein-coupled family of Kir channels (Hattori et al., 2000). This gene is located in the Down syndrome critical region, in the middle of the dual-specificity tyrosine phosphorylation-regulated kinase 1A (*DYRK1A*) and Down syndrome critical region gene 4 (*DSCR4*) genes (Hattori et al., 2000). The Kir3.2 channel mediates the inhibitory effect of G protein-coupled receptors required by neuromodulators and neurotransmitters, and thus controls neuronal excitability (Yamada et al., 1998; Mark and Herlitze, 2000). It is highly expressed in most mammalian tissues, where it participates in several physiological processes through coupling with other channel proteins, resulting in homo or hetero-multimeric complexes (Uhlen et al., 2005, 2010). Masotti et al. (2015) reported three cases with Keppen–Lubinsky syndrome, which is characterized by severe ID and multiple congenital anomalies. Genetic testing revealed the following mutations: in-frame heterozygous deletion of three nucleotides causing the loss of one amino acid (T152del) for two cases, and heterozygous missense mutation introducing the G154S amino acid change (Masotti et al., 2015). Animal models for in-frame deletions is presently not available and the mechanism underlying the above missense mutation is unknown.

Nevertheless, it has been demonstrated that an increase in *Kcnj6* gene dosage is essential for insufficiencies in behavior and synaptic plasticity of the dentate gyrus in the Ts65Dn murine model of Down syndrome (Kleschevnikov et al., 2017). The Kir3.2 channel blocker fluoxetine could rescue synaptic plasticity (Kleschevnikov et al., 2017), suggesting that gain-of-function property is likely the underlying mechanism; nevertheless, further studies are required to confirm that.

#### KCNJ10

*KCNJ10* (potassium channel inwardly rectifying subfamily J, member 10) encodes an ATP-sensitive inward rectifier potassium (Kir) channel comprising two putative transmembrane domains held together by an extracellular pore-forming region and flanked by amino and carboxy termini on the cytoplasmic side (Takumi et al., 1995). The KCNJ10 protein is present in glial cells of the CNS, predominantly in the cerebral cortex, cerebellar cortex, putamen, and caudate nucleus, as well as in renal epithelial cells and inner ear cells (Bockenhauer et al., 2009). Known also as Kir4.1, it is expressed in astrocytes and oligodendrocytes neighboring synapses and blood vessels, chiefly in the cortex, hippocampus, cerebellum, brainstem, spinal cord, thalamus, and olfactory bulb (Higashi et al., 2001; Hibino et al., 2004, 2010; Sicca et al., 2011).

*Mutations* in this gene have been demonstrated to reduce the channel's potassium current amplitude, surface expression, heteromer activity, and pH sensitivity (Bockenhauer et al., 2009; Hibino et al., 2010). These changes may alter the primary functions of astrocytes, including extracellular glutamate homeostasis, K^+^ siphoning, maintenance of resting membrane potential, and water volume regulation (Nwaobi et al., 2016). *KCNJ10* has been linked with EAST/SeSAME syndrome, which is characterized by epilepsy, ataxia, tubulopathy, sensorineural hearing loss, and occasionally ID (Celmina et al., 2019). Loss-of-function mutations are associated with mild ID (Scholl et al., 2009); whereas gain-of-function mutations correlate with mild to severe ID, depending on the variant (Sicca et al., 2011). The majority of reported mutations are of the loss-of-function type (Supplementary Table 1).

Sibille et al. (2014) showed that astroglial potassium clearance played a role in creating short-term plasticity of the tripartite synapse. Consequently, glial conditional *Kir4.1* KO mice demonstrated that astroglial potassium uptake decreased synaptic plasticity (Sibille et al., 2014). The authors concluded that astrocytes participated in synaptic activity through numerous channels and transporters and contributed to short-term plasticity partly through removal of K^+^ via Kir4.1 channels (Sibille et al., 2014). Likewise, conditional KO of hippocampus- *Kir4.1* led to inhibition of potassium and glutamate uptake, glial membrane depolarization, and heightened short-term synaptic potentiation (Djukic et al., 2007). Conditional KO of oligodendrocytes-*Kcnj10* resulted in late-onset axonal degeneration and mitochondrial damage associated with neuronal loss and neuro-axonal dysfunction (Schirmer et al., 2018). Mice deficient in oligodendrocyte Kir4.1 channels showed slow clearance of extracellular K^+^, delayed axonal recovery following repetitive stimulation in white matter, as well as low seizure threshold and motor deficits (Larson et al., 2018).

#### KCNJ11

*KCNJ11* (potassium voltage-gated channel subfamily J member 11) encodes the Kir6.2 subunit of the pancreatic ATP-sensitive potassium (KATP) channel (Nichols and Lopatin, 1997; Aguilar-Bryan and Bryan, 1999). This channel has two important subunits: the pore-forming Kir6.2 subunit and the sulfonylurea receptor 1 regulatory subunit (Clement et al., 1997). Its expression is elevated in beta cells and neurons (Nichols and Lopatin, 1997; Aguilar-Bryan and Bryan, 1999). An increased KATP current caused by channel opening results in membrane hyperpolarization in beta cells and consequent inhibition of insulin secretion (Bennett et al., 2010). Conversely, closure of this channel in reaction to augmented glucose triggers the release of insulin from beta cells into the bloodstream, thus helping to control blood sugar levels (Bennett et al., 2010). In neurons, an increased KATP current results in reduced electrical activity (Fendler et al., 2013).

*KCNJ11* mutations are associated with two major phenotypes: neonatal diabetes with moderate developmental delay and/or muscle weakness but not epilepsy (I-DEND) and developmental delay, epilepsy, and neonatal diabetes (DEND) (Flanagan et al., 2006; Mohamadi et al., 2010; Shah et al., 2012; Fendler et al., 2013; Lin et al., 2013; Carmody et al., 2016). Several variants with gain-of-function properties have been associated with ID: V59M, Y330C, V59A, G53D, R201C, C166Y, V59G, and Q52R. Most cases carry the V59M variant Supplementary Table 1, which shows clear association with ID symptoms (Svalastoga et al., 2020). Notably, Lin et al. (2013) reported a case with gain-of-function derived from the S225T mutation and P226_P232del, suggesting that both gain- and loss-of-function mutations could lead to I-DEND. Sulfonylureas act as inhibitors of KATP channels and represent an optimum treatment for diabetes and they can ameliorate the related neurological disorders (Pearson et al., 2006). However, other studies have not detected any benefits of sulfonylureas on neurological symptoms (Sagen et al., 2004; Klupa et al., 2005; Svalastoga et al., 2020), suggesting that sulfonylureas can partially cross the blood–brain barrier, and that the improved neurological symptoms observed resulted from increased cerebellar perfusion (Fendler et al., 2013). Thus, ID could be initiated from gain-of-function or as a complication of neonatal diabetes or due to unknown mechanisms associated with KATP channel, which the partial effects of sulfonylureas on neurological symptoms cannot confirm. The pathways involved to fully restore KATP channel function in other tissues might differ from those in the CNS (Bowman et al., 2019). Animal studies will help to determine which of these is most likely.

### Voltage-Gated Potassium Channels

Voltage-gated potassium (Kv) channels, the largest superfamily of potassium channels, are important for generating and relaying electrical impulses in the nervous system. By allowing the selective flow of K^+^ across neuronal membranes, they help establish the level of excitability and resting potential of the membrane, stimulate action potential waveforms and firing patterns, and regulate synaptic behavior (Ried et al., 1993). They are classified into 12 subfamilies.

#### KCNA2

*KCNA2* encodes the Kv1.2 channel, which is expressed widely in both the central and peripheral nervous systems (Vacher et al., 2008; Trimmer, 2015). Kv1.1, encoded by *KCNA1*, and Kv1.2 are co-expressed in big axons and are usually found in the same tetramers (Vacher et al., 2008; Trimmer, 2015). They contribute to the low-voltage-activated potassium current I Kv1. Kv1.2 channels are distributed in the distal axon initial segment and juxtaparanodes bordering the nodes of Ranvier (Trimmer, 2015). They mediate the D-type (delay) current (Storm, 1990; Grissmer et al., 1994; Brew et al., 2003), which is a critical controller of neuronal excitability as it actuates at subthreshold membrane potentials, rapidly deferring action potential commencement and averting repetitive firing (Storm, 1988, 1990). In addition, these channels work as delayed rectifiers, since blockage of Kv1.2 and D-type currents halts the surge in duration of action potentials (Kole et al., 2007; Shu et al., 2007), which is followed by increased calcium inflow in the presynaptic terminals and release of glutamate.

*KCNA2* mutations are related to various phenotypes including mild to severe ID/GDD and epilepsy (Syrbe et al., 2015; Hundallah et al., 2016), encephalopathy (Masnada et al., 2017), hereditary spastic paraplegias, mild ID, and ataxia (Helbig et al., 2016). Both gain- and loss-of-function mutations have been reported. Gain-of-function mutations such as L298F and R294H are linked to severe ID (Syrbe et al., 2015; Helbig et al., 2016). In contrast, loss-of-function mutations are associated with mild to moderate ID (Syrbe et al., 2015; Helbig et al., 2016). *Kcna2* KO mice revealed an important role of this channel in brain function, as gene deletion increased seizure susceptibility, reduced lifespan, and augmented the chance of premature death starting from the second week after birth (Brew et al., 2007). In a separate study, *Kcna2* KO mice demonstrated less non-rapid eye movement sleep and fewer seizures originating during such period (Douglas et al., 2007). Further studies will determine how both gain- and loss-of-function mutations lead to GDD/ID.

#### KCNA4

*KCNA4* encodes the Kv1.4 channel, also known as the Shaker-type potassium channel of the Kv channel family. The Kv1.4 channel is highly expressed in the striatal neurons, cerebral cortex, hippocampus, globus pallidus, cerebral peduncle, dorsal cochlear nuclei, and substantia nigra (Chung et al., 2000; Lujan et al., 2003), as well as in the retina (Holtje et al., 2007; Kaya et al., 2016). The Kv1.4 channel contributes to the generation of A-type K^+^ current channels in mature cortical pyramidal neurons (Carrasquillo et al., 2012) and the fast repolarizing phase of action potentials. A-type K^+^ currents play a central role in long-term plasticity, which is an essential mechanism for learning and memory (Chen et al., 2006).

Bauer et al. (2018) reported three cases that presented with facial dysmorphism, hypertrichosis, epilepsy, ID, and gingival overgrowth. Genetic testing revealed them to carry one of the following *de novo* mutations A172E, A244P, and A172E (Bauer et al., 2018). All three cases presented with severe ID and electrophysiological studies indicated gain-of-function as the underlying mechanism for this disorder (Bauer et al., 2018). Kaya et al. (2016) reported four cases from consanguineous family members, who presented with a novel disorder characterized by borderline ID, attention deficit hyperactivity disorder, striatal thinning, and congenital cataract. They all carried the R89Q variant (Kaya et al., 2016). Electrophysiological studies revealed loss-of-function as the mechanism responsible for this disorder (Kaya et al., 2016). Nevertheless, it remains unclear how *KCNA4* loss-of-function may lead to ID, thus further studies are warranted.

#### KCND3

*KCND3* (potassium voltage-gated channel, Shal-related subfamily, member 3) encodes the Kv4.3 channel, an alpha subunit of the Shal family of Kv channels (Serodio et al., 1996; Isbrandt et al., 2000). It is found in the brain and heart, and is important for membrane repolarization (Serodio et al., 1996; Isbrandt et al., 2000). Smets et al. (2015) reported a case with mild ID, epilepsy, attention deficit hyperactivity disorder, early onset cerebellar ataxia, strabismus, and oral apraxia. A *de novo* mutation (A293F295dup) was identified and the mechanism of disease was found to be haploinsufficiency (Smets et al., 2015). Kurihara et al. (2018) reported another case, this one carrying a *de novo* missense mutation (G384S). The clinical presentation consisted of mild ID, early onset cerebellar ataxia, myoclonus, and dystonia; however, no follow-up electrophysiological study was performed (Kurihara et al., 2018). What remains to be determined is how *KCND3* loss-of-function mutations may lead to ID.

#### KCNH1

*KCNH1* (potassium voltage-gated channel, subfamily H (eag related), member 1) encodes the voltage-gated Kv10.1 potassium channel, also called ether-a-go-go-related gene 1 (Gutman et al., 2005). The Kv10.1 channel is expressed in the cerebral cortex, hippocampus, cerebellum, and olfactory bulb (Ludwig et al., 1994, 2000; Gomez-Varela et al., 2010). Its presence in dopaminergic cells and the dentate gyrus of hippocampal neurons maintains electrophysiological activity patterns (Ferreira et al., 2012). It has an N-terminal Per-Arnt-Sim (PAS) domain and a C-terminal cyclic nucleotide-binding domain that control gating (James and Zagotta, 2018). Activation of this channel depends on the extracellular concentration of Mg^2+^ and on membrane potential (James and Zagotta, 2018).

Mutations in *KCNH1* are linked to Temple–Baraitser Syndrome, whose phenotype includes ID, epilepsy, and hypoplasia/aplasia of the nails of the thumb and great toe (Simons et al., 2015; Megarbane et al., 2016) and Zimmermann–Laband syndrome, which is characterized by ID, hypoplasia of nails and terminal phalanges, facial dysmorphism, gingival enlargement, and hypertrichosis (Kortum et al., 2015). In both conditions, cases present with severe to profound ID (Kortum et al., 2015; Simons et al., 2015; Megarbane et al., 2016). Reported variants include, G348R, G503R, L489F, I494V, K217N, I467V, S325Y, V356L, G469R, and L352V; all of them have gain-of-function channel properties (Kortum et al., 2015; Simons et al., 2015).

Zebrafish knockdown of *kcnh1* demonstrated severe neuronal developmental impairment, manifested as delayed hindbrain formation, growth retardation, and embryonic lethality (Stengel et al., 2012). Surprisingly, Kv10.1 null mice display normal memory, learning, social behavior, and sensorimotor functioning with mild hyperactivity (Ufartes et al., 2013). Bronk et al. (2018) showed that increased activity of the Kv10.1 channel in *Drosophila* resulted in lower presynaptic activity but higher postsynaptic activity through homeostatic plasticity. While the experiment attempted to explain the occurrence of epilepsy, it remains unclear how increased activity of the Kv10.1 channel and subsequent surge in postsynaptic activity could lead to severe forms of ID. Mortensen et al. (2015) reported that the Kv10.1 channel was more abundant in the presynaptic terminals and did not contribute to somatic action potentials; instead, it controlled Ca^2+^ influx and neurotransmitter discharge throughout repetitive high-frequency activity. Future studies using different Kv10.1 variant knock-in mice will help identify the mechanism by which Kv10.1 gain-of-function mutations lead to severe ID and epilepsy.

#### KCNQ2

*KCNQ2* encodes the Kv7.2 subfamily Q member 2 Kv channel. Kv7.2 channels are widely expressed in the brain, especially in the axon's initial segment and nodes of Ranvier (Greene and Hoshi, 2017). They trigger the M current, a voltage-gated non-inactivating potassium current. They control the firing of pyramidal neurons in one of the following ways: (1) help set the initial segment membrane potential; (2) contribute to the generation of medium afterhyperpolarization for refractory period; (3) regulate firing frequency, theta resonance, and transient neuronal hyperexcitability (Greene and Hoshi, 2017). The activation and possibility of opening this channel depend on phosphatidylinositol 4, 5-bisphosphate (PIP2) (Li et al., 2005; Kim et al., 2016). Although mutations of this gene are associated with early infantile epileptic encephalopathy, West syndrome, and Ohtahara syndrome, all of which are often accompanied by severe ID (Weckhuysen et al., 2012, 2013; Kato et al., 2013; Zhang Y. et al., 2015; Dimassi et al., 2016; Zhang et al., 2017), cases with ID related to childhood onset of seizures have also been reported (Borgatti et al., 2004; Hewson et al., 2017). Hewson et al. (2017) reported a three-generation pedigree (*n* = 6), with ID ranging from borderline to moderate caused by R210C loss-of-function mutation (Soldovieri et al., 2019). Borgatti et al. (2004) reported two cases with moderate to profound ID accompanied by benign familial neonatal convulsion focal seizures, which were ascribed to the K554N loss-of-function mutation. Most of those who present with early infantile epileptic encephalopathy have loss-of-function mutations (Orhan et al., 2014; Zhang et al., 2017); nevertheless, some cases with gain-of-function mutations have been reported, too. Miceli et al. (2015a) proved that R201C, R144Q, and R201H mutations identified in patients with epileptic encephalopathies and/or ID possessed gain-of-function characteristics. Millichap et al. (2017) reported a case presenting with infantile spasms and severe ID, without previous neonatal seizures, carrying the R198Q gain-of-function variant. Gain-of-function R201C and R201H variants were found in 10 cases with severe neonatal encephalopathy but devoid of neonatal seizures (Mulkey et al., 2017). The M-current blocker XE991 has been reported to improve learning and memory in healthy mice (Fontan-Lozano et al., 2011). Thus, gain-of-function mutations might explain ID. For epileptic encephalopathy cases, it remains unclear whether the severe to profound ID is a consequence of epileptic activity *per se*, because seizure frequency and electroencephalography abnormalities do not match the degree of ID and behavioral disturbances in patients with *SCN1A* mutations (Nabbout et al., 2013). Nevertheless, this link has not been studied in KCNQ2 epileptic disorders. Retigabine, a Kv7.2/Kv7.3-channel opener, diminishes KA-induced seizure activities in knock-in mice (Kcnq2Y284C/^+^ and Kcnq2A306T/^+^), was approved by the Food and Drug Administration as an anticonvulsant (Ihara et al., 2016), however, it was removed due to its side effects (Abou-Khalil, 2019). Thus, more studies are required to explore how gain-of-function mutations result in epileptic encephalopathies, as well as how loss-of-function mutations lead to ID.

#### KCNQ3

*KCNQ3* (potassium voltage-gated channel subfamily Q member 3) encodes the Kv7.3 voltage-gated ion channel subunit. This, together with Kv7.2, facilitates the M-current (IKM), which is vital for putting off neuronal excitability (Wang et al., 1998). Kv7.3 channels are common in the brain especially in the axon's initial segment and nodes of Ranvier (Greene and Hoshi, 2017). They control the firing of pyramidal neurons either by initiating the axon initial segment membrane potential, restricting the depolarization that follows an action potential or defining their input resistance during relaxation and as neurons come near the action potential threshold (Greene and Hoshi, 2017). The activation and possibility of opening this channel depend on PIP2 (Li et al., 2005; Kim et al., 2016).

Gain-of-function mutations R230C and R227Q have been reported in cases presenting with moderate to severe ID (Sands et al., 2019); however, the same phenotype has been associated also with the loss-of-function mutation R330L (Miceli et al., 2015b). Hence, it remains to be determined how both types of mutations in *KCNQ3* can lead to mild to severe ID. The M-current blocker XE991 has been demonstrated to improve learning and memory in healthy mice (Fontan-Lozano et al., 2011).

#### KCNQ5

*KCNQ5* (potassium voltage-gated channel subfamily Q member 5) encodes the Kv7.5 channel (Schroeder et al., 2000). This channel plays a key role in regulating M-type current and afterhyperpolarization conductance, resulting in neuronal excitability. It is highly expressed in the brain, especially in presynaptic terminals of pyramidal neurons and hippocampal interneurons, which enables the regulation of inhibitory inputs within the hippocampal network (Fidzinski et al., 2015). Lehman et al. (2017) reported three cases with mild to severe ID accompanied with epilepsy and characterized by both loss-of-function mutations, such as S448I, V145G, L341I, as well as the gain-of-function mutation P369R in one case that presented with severe to profound ID. Rosti et al. (2019) reported a case presenting with mild ID and epilepsy, for whom genetic tests revealed V133^*^. The molecular mechanism underlying this condition was haploinsufficiency. A dominant-negative *Kcnq5* mutation in mice showed alteration of synaptic inhibition and excitability in the hippocampus; however, no seizures were detected (Tzingounis et al., 2010; Fidzinski et al., 2015). Because the M-current blocker XE991 enhances learning and memory in healthy mice (Fontan-Lozano et al., 2011), it is unclear how both gain- and loss-of-function mutations in *KCNQ5* can lead to ID.

#### KCNAB1

*KCNAB1* (potassium voltage-gated channel subfamily A member regulatory beta subunit 1) encodes the Kvbeta1 Shaker-related channel (Butler et al., 1998). Kvbeta1 channels are particularly abundant in the cerebral cortex, hippocampus, cerebellum, dorsal striatum, and colliculus (Butler et al., 1998). They control action potentials through their effect on pore-forming alpha subunits and facilitate closing of delayed rectifier potassium channels by using their N-terminal domain to block the pore (Accili et al., 1998). This ultimately makes it easier for other members of the same channel family to close as fast as possible (Accili et al., 1998). Zhang Y. et al. (2015) reported a case with early onset epileptic encephalopathy with severe ID. Genetic testing revealed *de novo* mutations in *KCNAB1* (L355Hfs^*^5), but were not complemented by electrophysiological studies. Murphy et al. (2004) demonstrated improved learning, neuronal excitability, and synaptic plasticity in Kvbeta1.1 KO mice. The Kvbeta1.1-deficient mice exhibited usual synaptic plasticity but displayed impaired learning of a water maze test and in the social transmission of food preference task, signifying that the Kvbeta1.1 subunit contributes to certain kinds of learning and memory (Giese et al., 1998). Thus, it remains unclear how *KCNAB1* mutations lead to ID, and hence further investigations are warranted.

#### KCNC1

*KCNC1* (potassium voltage-gated channel subfamily C member 1) encodes the Kv3.1 channel, a subunit of the Kv3 subfamily of channels. They are abundant in the CNS, particularly in GABAergic interneurons (Gan and Kaczmarek, 1998). These channels are critical constituents of the circuitry of neurons as they can fire action potentials at high frequency (Wang et al., 2007) and are among the channels regulated by FMRP (Strumbos et al., 2010).

Loss of Kv3 function interrupts the firing of fast-spiking neurons and disturbs the release of neurotransmitter (Sabatini and Regehr, 1997; Erisir et al., 1999; Issa et al., 2011). Poirier et al. (2017) reported three cases with moderate to severe ID carrying the loss-of-function mutation R339^*^ while a similar case, T399M, was reported recently by Park et al. (2019) and six cases were reported by Cameron et al. (2019), including mutations A421V, R317H, and Q492X.

Thus, it seems that both ID and epilepsy can occur due to loss-of-function mutations, although the exact mechanism underlying this process will require further studies. Mice lacking Kv3.1 channels demonstrated altered synaptic transmission and motor dysfunction, suggesting a role for this channel in cognition (Matsukawa et al., 2003). However, other studies showed that Kv3.1- deficient mice had no obvious learning or memory deficit, only motor dysfunction (Espinosa et al., 2001; Zhang and Kaczmarek, 2016). Kv3.1 channel modulator (AUT000206) could rescue cognitive deficits in a schizophrenia phencyclidine model (Reynolds and Neill, 2016).

#### KCNC3

*KCNC3* (potassium voltage-gated channel subfamily C member 3) encodes the Kv channel Kv3.3 subunit (Gutman et al., 2005). Its major purpose is to initiate the repolarization phase of action potentials (Rudy and McBain, 2001). These channels are abundant in the brain stem and cerebellum (Rudy and McBain, 2001; Chang et al., 2007; Puente et al., 2010). Duarri et al. (2015) reported three cases, of which one presented with severe ID and two with mild ID accompanied with cerebellar ataxia. They had the following mutations: sporadic D129N (gain-of-function) for the case with severe ID, sporadic R423H (loss-of-function) and sporadic and familial V535M (gain-of-function) for the mild ID cases. How gain-of-function mutations can result in both severe and mild ID needs to be explored further. Mice lacking Kv3.3 channels demonstrated altered synaptic transmission and motor dysfunction, suggesting a role in cognition (Matsukawa et al., 2003). However, another study showed that Kv3.3-deficient mice had no obvious learning or memory deficit but displayed motor dysfunction (Espinosa et al., 2001; Zhang and Kaczmarek, 2016).

#### KCNB1

*KCNB1* (potassium voltage-gated channel subfamily B member 1) encodes the Kv2.1 channel potentials (Guan et al., 2013; Liu and Bean, 2014). Kv2.1 channels produce delayed-rectifier potassium currents in hippocampal and cortical pyramidal neurons, and are essential for afterhyperpolarization by their action potentials (Guan et al., 2013; Liu and Bean, 2014). They are abundant in the proximal axon initial segment (Trimmer, 2015) and can result in either excitatory or inhibitory neuronal activity depending on the extent of the stimulus (Liu and Bean, 2014). Torkamani et al. (2014) reported three cases with severe ID accompanied by epileptic encephalopathy. All had mutations with loss of function effect, such as *de novo* missense mutations S347R, G379R, and T374I. Srivastava et al. (2018) reported two cases with severe ID diagnosed as atypical Rett syndrome. Genetic studies revealed their mutations to be G379R and T374I (loss of function effect). In addition, Krey et al. (2019) recently reported a case diagnosed with both severe ID and West syndrome, which was revealed to originate from the *de novo* heterozygous deletion mutation W370^*^. Additional variants with loss-of-function properties detected in cases with severe GDD/ID include R583^*^, K502fs, G401R, V378A, and R306C (Saitsu et al., 2015; Thiffault et al., 2015; de Kovel et al., 2017). Thus, it seems like loss-of-function mutations of *KCNB1* may lead to ID.

Mutant Kv2.1(^−^/^−^) mice deficient for the Kv2.1 channel demonstrated decreased long-term potentiation at the Schaffer collateral-CA1 synapse, impaired spatial learning, failure to perform better in a Morris water maze, and hyperactivity (Speca et al., 2014). Besides, these mice were not prone to spontaneous seizures but rather exhibited enhanced seizure advancement. Kv2.1 is transferred to membrane-bound clusters, which are detected in rodent dopamine neurons both *in vivo* and *in vitro* (Lebowitz et al., 2019). This finding suggests that an altered functional interaction between the dopamine transporter and Kv2.1 impacts dopamine neuron activity (Lebowitz et al., 2019).

#### KCTD3

*KCTD3* (potassium channel tetramerization domain containing 3) encodes a protein found to bind to the hyperpolarization-activated cyclic nucleotide-gated channel HCN3 (Cao-Ehlker et al., 2013). There are few studies regarding the function of this gene. Kctd3 and Hcn3 co-localize in the cerebellum, hypothalamus, and midbrain and Kctd3 upregulates the expression of Hcn3 (Cao-Ehlker et al., 2013). HCN3 modulates synaptic strength and cellular excitability (Biel et al., 2009; Huang et al., 2011). Faqeih et al. (2018) reported seven cases with severe GDD, epilepsy, and cerebellar hypoplasia, of which five were carrying P346Tfs^*^4 and the other two were carrying R56^*^ mutations. Electrophysiological studies revealed loss-of-function as a mechanism underlying the disease (Faqeih et al., 2018). Similarly, Alazami et al. (2015) and Trujillano et al. (2017) reported two cases with severe GDD, epilepsy, and cerebellar hypoplasia, who carried the P346Tfs^*^4 variant. Thus, it seems *KCTD3* loss-of-function is the underlying mechanism for GDD/ID. However, more animal model studies are needed to confirm this hypothesis.

## Discussion

Intellectual disability (ID) limits intellectual functioning and adaptive behavior since an early age. Potassium channels have diverse gating properties and are often involved in learning and memory. This review shows that potassium channelopathies can play an important role in initiating ID, with both gain- and loss-of-function mutations leading to ID. We report that of the nineteen identified channelopathies, more than half are characterized by both gain- and loss-of-function gene mutations, and for many no suitable animal models exist. Moreover, available channel blockers or openers offer only modest benefits to patients. Likewise, a total number of twelve potassium channelopathies have been reported to associate with epilepsy, and some of them have both gain- and loss-of-function properties (Niday and Tzingounis, 2018; Allen et al., 2020).

Recent studies have given insights into how to approach channelopathies in other conditions besides ID. Strategies that are currently used include gene therapy and/or gene editing (Choong et al., 2016; Collins and Gottlieb, 2018; Wykes and Lignani, 2018; Shahi et al., 2019). Approaches for gene therapies aim to modulate neuronal excitability, increase inhibitory tone, manipulate the expression levels of channels, as well as optogenetics and chemogenetics (Wykes and Lignani, 2018). Since Ginn's et al., paper in 2018, 2,600 gene therapy clinical trials were completed/ongoing/approved globally. Results have indicated that gene therapy is safe and effective (Ginn et al., 2018; Wykes and Lignani, 2018). However, it faces some challenges which need to be addressed such as lack of efficient methods for gene delivery and cell-mediated destruction of the gene-corrected cells (Ginn et al., 2018; Wykes and Lignani, 2018). Wykes and Lignani (2018) summarized strategies that can be utilized to deliver gene therapies into neurons including lentivirus, adeno-associated viruses, promoters, focal/global viral-mediated delivery of transgenes or CRISPR-Cas and controlling transgene expression. Likewise, gene therapy/editing can be utilized in ID.

Despite the fact that we collected information regarding the total number of cases reported, we did not focus on them. We could not discuss the relationship between ID and other channelopathies (sodium, calcium, and chloride) as it was beyond the capacity of one article. This review is limited by the lack of available information on distinct animal models allowing to describe the specific role of each reported gene during brain development and their specific modulators.

## Conclusions

Potassium channelopathies contribute to the occurrence of ID in several cases. A total of 19 channelopathies are known so far, affecting the following genes: *KCNMA1, KCNN3, KCNT1, KCNT2, KCNJ10, KCNJ6, KCNJ11, KCNA2, KCNA4, KCND3, KCNH1, KCNQ2, KCNAB1, KCNQ3, KCNQ5, KCNC1, KCNB1, KCNC3*, and *KCTD3*. Both gain- and loss-of-function mutations are associated with GDD/ID. The mechanisms of how both gain- and loss-of-function mutations lead to ID are unknown to a large extent. There is a paucity of animal studies on the mechanisms of ID in relation to potassium channelopathies. Some of the few available treatment options (channel openers or blockers) have demonstrated limited benefits in clinical settings.

## Recommendations

Future studies should focus on understanding the effect of gain- and loss-of-function mutations in neurons responsible for learning and memory. Those studies should go further to explore the interaction between each specific channel and other proteins, which might also play a role in cognition, as suggested by the modest effect of available channel blockers or openers. This knowledge will aid in identifying new targets and developing new treatments for ID related to potassium channelopathies; gene therapies/editing.

## Data Availability Statement

All datasets generated for this study are included in the article/Supplementary Material.

## Author Contributions

MK and BC are first co-authors who designed study, reviewed the articles, drafted, and wrote the manuscript. YT and EO assisted in preparing table and figure. JP, FH, LY, and FY revised the manuscript and supervised each step involved in the preparation of the manuscript. All co-authors have read and agreed to the content of the manuscript.

## Conflict of Interest

The authors declare that the research was conducted in the absence of any commercial or financial relationships that could be construed as a potential conflict of interest.
