# Supplementary material for: Intellectual Disability and Potassium Channelopathies: A Systematic Review
Source: Front Genet. 2020 Jun 23;11:614. doi: 10.3389/fgene.2020.00614 (PMC7324798; doi:10.3389/fgene.2020.00614)
Supplement: Supplementary file 1 [file Table_1.docx]

**Supplementary Table 1**

**Potassium channel gene mutations reported to associate with global developmental delay/intellectual disability**

| **Name of the gene** | **Syndrome/Phenotype** | **OMIM number** | **Protein change** | **Type of mutation** | **Electrophysiological study result** | **Severity of the GDD/ID** | **Reference** |
| --- | --- | --- | --- | --- | --- | --- | --- |
| *KCNN3* | ZLS | 602983 | S436C | Missense | GOF | Moderate ID | Bauer et al., 2019 |
| *KCNN3* | ZLS | 602983 | K269E | Missense | GOF | GDD | Bauer et al., 2019 |
| *KCNN3* | ZLS | 602983 | G350D | Missense | GOF | GDD | Bauer et al., 2019 |
| *KCNJ10* | ID, EP and ASD | 602208 | R18Q | Missense | GOF | Moderate ID | Sicca et al., 2011 |
| *KCNJ10* | ID, EP and ASD | 602208 | R18Q | Missense | GOF | Mild ID | Sicca et al., 2011 |
| *KCNJ10* | ID, EP and ASD | 602208 | V84M | Missense | GOF | Severe ID | Sicca et al., 2011 |
| *KCNJ10* | EP and ID | 602208 | R65C and F119GfsX25 | Missense and frameshift | Unknown | Mild ID | Papavasiliou et al., 2017 |
| *KCNJ10* | EP and ID | 602208 | I60T | Missense | Unknown | Moderate ID | Al Dhaibani et al., 2018 |
| *KCNJ10* | EP and ID | 602208 | I60T | Missense | Unknown | Moderate ID | Al Dhaibani et al., 2018 |
| *KCNJ10* | EAST syndrome | 602208 | N232Qfs*14 | Nonsense | LOF | Moderate ID | Severino et al., 2018 |
| *KCNJ10* | EAST syndrome | 602208 | G275Vfs*7 | Nonsense | LOF | Moderate ID | Severino et al., 2018 |
| *KCNJ10* | SeSAME syndrome | 602208 | R65P and R199* | Compound missense and nonsense | LOF | Mild ID | Scholl et al., 2009 |
| *KCNJ10* | SeSAME syndrome | 602208 | C140R | Missense | LOF | Mild ID | Scholl et al., 2009 |
| *KCNJ10* | SeSAME syndrome | 602208 | T164I | Missense | LOF | Mild ID | Scholl et al., 2009 |
| *KCNJ10* | SeSAME syndrome | 602208 | A167V and R297C | Compound missense | LOF | Mild ID | Scholl et al., 2009 |
| *KCNJ10* | ID and EP, truncal ataxia and dysmorphic features and electrolyte imbalance | 602208 | R204H | Missense | Unknown | Severe ID | Kara et al., 2013 |
| *KCNA2* | Severe GDD and EP | 176262 | T374A | Missense | LOF | Severe GDD | Hundallah et al., 2016 |
| *KCNA2* | Hereditary spastic paraplegia, ID and ataxia. | 176262 | R294H | Missense | LOF | Mild ID | Helbig et al., 2016 |
| *KCNA2* | Hereditary spastic paraplegia, ID and ataxia. | 176262 | R294H | Missense | LOF | Mild ID | Helbig et al., 2016 |
| *KCNA2* | Hereditary spastic paraplegia, ID and ataxia. | 176262 | R294H | Missense | LOF | Mild ID | Helbig et al., 2016 |
| *KCNA2* | Hereditary spastic paraplegia, ID and ataxia. | 176262 | R294H | Missense | LOF | Mild ID | Helbig et al., 2016 |
| **Name of the gene** | **Syndrome/Phenotype** | **OMIM number** | **Protein change** | **Type of mutation** | **Electrophysiological study result** | **Severity of the GDD/ID** | **Reference** |
| *KCNA2* | Hereditary spastic paraplegia, ID and ataxia. | 176262 | R294H | Missense | LOF | Mild ID | Helbig et al., 2016 |
| *KCNA2* | EP and ID | 176262 | P405L | Nonsense | LOF | Moderate ID | Syrbe et al., 2015 |
| *KCNA2* | EP and ID | 176262 | I263T | Nonsense | LOF | Moderate ID | Syrbe et al., 2015 |
| *KCNA2* | EP and ID | 176262 | P405L | Nonsense | LOF | ID | Syrbe et al., 2015 |
| *KCNA2* | EP and ID | 176262 | P405L | Nonsense | LOF | Moderate ID | Syrbe et al., 2015 |
| *KCNA2* | EP and ID | 176262 | L298F | Missense | GOF | Severe ID | Syrbe et al., 2015 |
| *KCNA2* | EP and ID | 176262 | R297Q | Missense | GOF | Moderate ID | Syrbe et al., 2015 |
| *KCNA4* | ID, striatal thinning, congenital  cataract and ADHD | 176266 | R89Q | Missense | LOF | Mild ID | Kaya et al., 2016 |
| *KCNA4* | ID, striatal thinning, congenital  cataract and ADHD | 176266 | R89Q | Missense | LOF | Mild ID | Kaya et al., 2016 |
| *KCNA4* | ID, striatal thinning, congenital  cataract and ADHD | 176266 | R89Q | Missense | LOF | Mild ID | Kaya et al., 2016 |
| *KCNA4* | ID, striatal thinning, congenital  cataract and ADHD | 176266 | R89Q | Missense | LOF | Mild ID | Kaya et al., 2016 |
| *KCNA4* | FHEIG | 176266 | A172E | Missense | GOF | Severe ID | Bauer et al., 2018 |
| *KCNA4* | FHEIG | 176266 | A244P | Missense | GOF | Moderate ID | Bauer et al., 2018 |
| *KCNA4* | FHEIG | 176266 | A172E | Missense | GOF | Severe ID | Bauer et al., 2018 |
| *KCND3* | Cerebellar ataxia complicated by ID, EP, and ADHD | 605411 | A293F295dup | Duplication | LOF | Mild ID | Smets et al., 2015 |
| *KCND3* | ID, ataxia, myoclonus, and dystonia. | 605411 | G384S | Missense | Unknown | Mild ID | Kurihara et al., 2018 |
| *KCNH1* | TBS | 603305 | G348R | Missense | GOF | Severe ID | Megarbane et al., 2016 |
| *KCNH1* | TBS | 603305 | G503R | Missense | GOF | Severe ID | Simons et al., 2015 |
| *KCNH1* | TBS | 603305 | L489F | Missense | GOF | Severe ID | Simons et al., 2015 |
| *KCNH1* | TBS | 603305 | I494V | Missense | GOF | Severe ID | Simons et al., 2015 |
| *KCNH1* | TBS | 603305 | K217N | Missense | GOF | Severe ID | Simons et al., 2015 |
| *KCNH1* | TBS | 603305 | I494V | Missense | GOF | Severe ID | Simons et al., 2015 |
| *KCNH1* | TBS | 603305 | I494V | Missense | GOF | Severe ID | Simons et al., 2015 |
| **Name of the gene** | **Syndrome/Phenotype** | **OMIM number** | **Protein change** | **Type of mutation** | **Electrophysiological study result** | **Severity of the GDD/ID** | **Reference** |
| *KCNH1* | ZLS | 603305 | I467V | Missense | GOF | Severe ID | Kortum et al., 2015 |
| *KCNH1* | ZLS | 603305 | S325Y and V356L | Missense | Both were GOF | Profound ID | Kortum et al., 2015 |
| *KCNH1* | ZLS | 603305 | G348R | Missense | GOF | Profound ID | Kortum et al., 2015 |
| *KCNH1* | ZLS | 603305 | G469R | Missense | GOF | Mild ID | Kortum et al., 2015 |
| *KCNH1* | ZLS | 603305 | L352V | Missense | GOF | Severe ID | Kortum et al., 2015 |
| *KCNH1* | ZLS | 603305 | I467V | Missense | GOF | Severe ID | Kortum et al., 2015 |
| *KCNQ2* | ID, EP and behavioral disorder | 602235 | R210C | Missense | LOF | Moderate ID | Hewson et al., 2017 |
| *KCNQ2* | EP and moderate ID | 602235 | R210C | Missense | LOF | Moderate ID | Hewson et al., 2017 |
| *KCNQ2* | EP and moderate ID | 602235 | R210C | Missense | LOF | Moderate ID | Hewson et al., 2017 |
| *KCNQ2* | EP and moderate ID | 602235 | R210C | Missense | LOF | Moderate ID | Hewson et al., 2017 |
| *KCNQ2* | EP and moderate ID | 602235 | R210C | Missense | LOF | Moderate ID | Hewson et al., 2017 |
| *KCNQ2* | EP and moderate ID | 602235 | R210C | Missense | LOF | Moderate ID | Hewson et al., 2017 |
| *KCNQ2* | Epileptic encephalopathy | 602235 | E140Q | Missense | LOF | Moderate GDD | Soldovieri et al., 2019 |
| *KCNQ2* | ID and EP | 602235 | R213Q | Missense | LOF | Profound ID | Weckhuysen et al., 2012 |
| *KCNQ2* | ID and EP | 602235 | T274M | Missense | LOF | Profound ID | Weckhuysen et al., 2012 |
| *KCNQ2* | ID and EP | 602235 | I205V | Missense | LOF | Moderate ID | Weckhuysen et al., 2012 |
| *KCNQ2* | ID and EP | 602235 | R560W | Missense | LOF | Profound ID | Weckhuysen et al., 2012 |
| *KCNQ2* | ID and EP | 602235 | M265V | Missense | LOF | Profound ID | Weckhuysen et al., 2012 |
| *KCNQ2* | ID and EP | 602235 | M546V | Missense | LOF | Profound ID | Weckhuysen et al., 2012 |
| *KCNQ2* | ID and EP | 602235 | G290D | Missense | LOF | Profound ID | Weckhuysen et al., 2012 |
| *KCNQ2* | ID and EP | 602235 | G290D | Missense | LOF | Profound ID | Weckhuysen et al., 2012 |
| *KCNQ2* | West syndrome | 602235 | Y284H | Missense | LOF | Severe ID | Zhang et al., 2017 |
| *KCNQ2* | West syndrome | 602235 | R291G | Missense | LOF | Severe ID | Zhang et al., 2017 |
| **Name of the gene** | **Syndrome/Phenotype** | **OMIM number** | **Protein change** | **Type of mutation** | **Electrophysiological study result** | **Severity of the GDD/ID** | **Reference** |
| *KCNQ2* | West syndrome | 602235 | Y237F | Missense | LOF | Severe ID | Zhang et al., 2017 |
| *KCNQ2* | Non syndromic | 602235 | G290S | Missense | LOF | Severe ID | Zhang et al., 2017 |
| *KCNQ2* | West syndrome | 602235 | Y280H | Missense | LOF | Severe ID | Zhang et al., 2017 |
| *KCNQ2* | Ohtahara syndrome | 602235 | E484D | Missense | LOF | Severe ID | Zhang et al., 2017 |
| *KCNQ2* | West syndrome | 602235 | Q429Rfs*5 | Nonsense | LOF | Severe ID | Zhang et al., 2017 |
| *KCNQ2* | West syndrome | 602235 | F305L | Missense | LOF | Severe ID | Zhang et al., 2017 |
| *KCNQ2* | West syndrome | 602235 | A246P | Missense | LOF | Severe ID | Zhang et al., 2017 |
| *KCNQ2* | West syndrome | 602235 | A265T | Missense | LOF | Severe ID | Zhang et al., 2017 |
| *KCNQ2* | Non syndromic | 602235 | V250L | Missense | LOF | Severe ID | Zhang et al., 2017 |
| *KCNQ2* | West syndrome | 602235 | T274M | Missense | LOF | Severe ID | Zhang et al., 2017 |
| *KCNQ2* | Non syndromic | 602235 | R213W | Missense | LOF | Severe ID | Zhang et al., 2017 |
| *KCNQ2* | Ohtahara syndrome | 602235 | T217N | Missense | GOF | Profound ID | Kato et al., 2013 |
| *KCNQ2* | EOEE, unclassified | 602235 | A265V | Missense | Unknown | Moderate ID | Kato et al., 2013 |
| *KCNQ2* | Ohtahara syndrome | 602235 | A265V | Missense | Unknown | Profound ID | Kato et al., 2013 |
| *KCNQ2* | Ohtahara syndrome | 602235 | P285H | Missense | Unknown | Moderate ID | Kato et al., 2013 |
| *KCNQ2* | EOEE, unclassified | 602235 | G290S | Missense | Unknown | Profound ID | Kato et al., 2013 |
| *KCNQ2* | Ohtahara syndrome | 602235 | A294V | Missense | LOF | Profound ID | Kato et al., 2013 |
| *KCNQ2* | Ohtahara syndrome | 602235 | A294V | Missense | LOF | Profound ID | Kato et al., 2013 |
| *KCNQ2* | Ohtahara syndrome | 602235 | R333W | Missense | Unknown | Severe MR | Kato et al., 2013 |
| *KCNQ2* | Ohtahara/West syndrome | 602235 | R553W | Missense | Unknown | Profound ID | Kato et al., 2013 |
| *KCNQ2* | Ohtahara syndrome | 602235 | R553L | Missense | Unknown | Profound ID | Kato et al., 2013 |
| *KCNQ2* | Ohtahara syndrome | 602235 | P561L | Missense | Unknown | Profound ID | Kato et al., 2013 |
| *KCNQ2* | Ohtahara syndrome | 602235 | D563E | Missense | Unknown | Moderate ID | Kato et al., 2013 |
| **Name of the gene** | **Syndrome/Phenotype** | **OMIM number** | **Protein change** | **Type of mutation** | **Electrophysiological study result** | **Severity of the GDD/ID** | **Reference** |
| *KCNQ2* | EOEE | 602235 | G315R | Missense | Unknown | Severe ID | Weckhuysen et al., 2013 |
| *KCNQ2* | EOEE | 602235 | G281R | Missense | Unknown | Moderate ID | Weckhuysen et al., 2013 |
| *KCNQ2* | EOEE | 602235 | R210H | Missense | Unknown | GDD | Weckhuysen et al., 2013 |
| *KCNQ2* | EOEE | 602235 | D563N | Missense | LOF | GDD | Weckhuysen et al., 2013; Ambrosino et al., 2018b |
| *KCNQ2* | EOEE | 602235 | F305L | Missense | Unknown | Moderate ID | Weckhuysen et al., 2013 |
| *KCNQ2* | EOEE | 602235 | S195P | Missense | Unknown | Profound ID | Weckhuysen et al., 2013 |
| *KCNQ2* | EOEE | 602235 | G315R | Missense | Unknown | Profound ID | Weckhuysen et al., 2013 |
| *KCNQ2* | EOEE | 602235 | R201C | Missense | GOF | Profound ID | Weckhuysen et al., 2013 |
| *KCNQ2* | EOEE | 602235 | A265T | Missense | Unknown | Profound ID | Weckhuysen et al., 2013 |
| *KCNQ2* | EOEE | 602235 | R325G | Missense | LOF | Profound ID | Weckhuysen et al., 2013 |
| *KCNQ2* | EOEE | 602235 | R325G | Missense | LOF | Profound ID | Weckhuysen et al., 2013 |
| *KCNQ2* | EOEE | 602235 | R325G | Missense | LOF | Profound GDD | Soldovieri et al., 2016 |
| *KCNQ2* | EOEE | 602235 | R325G | Missense | LOF | Profound GDD | Soldovieri et al., 2016 |
| *KCNQ2* | EOEE | 602235 | R325G | Missense | LOF | Profound GDD | Soldovieri et al., 2016 |
| *KCNQ2* | EOEE | 602235 | R325G | Missense | LOF | Profound GDD | Soldovieri et al., 2016 |
| *KCNQ2* | EOEE | 602235 | G281R | Missense | Unknown | Profound ID | Weckhuysen et al., 2013 |
| *KCNQ2* | EOEE | 602235 | R210H | Missense | Unknown | Moderate ID | Weckhuysen et al., 2013 |
| *KCNQ2* | EOEE | 602235 | R210H | Missense | Unknown | Moderate ID | Weckhuysen et al., 2013 |
| *KCNQ2* | EOEE | 602235 | K552T | Missense | Unknown | Moderate ID. | Weckhuysen et al., 2013 |
| *KCNQ2* | EOEE | 602235 | D563N | Missense | Unknown | Severe ID | Weckhuysen et al., 2013 |
| *KCNQ2* | EOEE | 602235 | K556E | Missense | Unknown | Moderate ID | Weckhuysen et al., 2013 |
| *KCNQ2* | EIEE | 602235 | S122L | Missense | LOF | Severe ID | Zhang Y. et al., 2015 |
| *KCNQ2* | EIEE | 602235 | K319T | Missense | Unknown | Severe ID | Zhang Y. et al., 2015 |
| **Name of the gene** | **Syndrome/Phenotype** | **OMIM number** | **Protein change** | **Type of mutation** | **Electrophysiological study result** | **Severity of the GDD/ID** | **Reference** |
| *KCNQ2* | EIEE | 602235 | T277I | Missense | Unknown | Severe ID | Zhang Y. et al., 2015 |
| *KCNQ2* | EIEE | 602235 | K552T | Missense | Unknown | Severe ID | Zhang Y. et al., 2015 |
| *KCNQ2* | West syndrome | 602235 | A306T | Missense | Unknown | Severe ID | Dimassi et al., 2016 |
| *KCNQ2* | ID and benign familial neonatal convulsion | 602235 | K554N | Missense | LOF | Profound ID | Borgatti et al., 2004 |
| *KCNQ2* | ID and benign familial neonatal convulsion | 602235 | K554N | Missense | LOF | Moderate ID | Borgatti et al., 2004 |
| *KCNQ2* | EOEE | 602235 | A294V | Missense | LOF | GDD | Abidi et al., 2015 |
| *KCNQ2* | EOEE | 602235 | A294V | Missense | LOF | GDD | Abidi et al., 2015 |
| *KCNQ2* | EOEE | 602235 | A294V | Missense | LOF | GDD | Abidi et al., 2015 |
| *KCNQ2* | EOEE | 602235 | A294V | Missense | LOF | GDD | Abidi et al., 2015 |
| *KCNQ2* | EOEE | 602235 | A294V | Missense | LOF | GDD | Abidi et al., 2015 |
| *KCNQ2* | EOEE | 602235 | A294V | Missense | LOF | Severe GDD | Abidi et al., 2015 |
| *KCNAB1* | EIEE | 601141 | L355Hfs*5 | Nonsense | LOF | Severe ID | Zhang Y. et al., 2015 |
| *KCNQ3* | ID and EP | 602232 | R330L | Missense | LOF | Mild ID | Miceli et al., 2015b |
| *KCNQ3* | ID and EP | 602232 | R330L | Missense | LOF | Severe ID | Miceli et al., 2015b |
| *KCNQ3* | ID and EP | 602232 | R230L | Missense | Unknown | Mild ID | Yamamoto et al., 2019 |
| *KCNQ3* | ID | 602232 | R230C | Missense | GOF | Moderate ID | Sands et al., 2019 |
| *KCNQ3* | ID and ASD | 602232 | R230C | Missense | GOF | Severe ID | Sands et al., 2019 |
| *KCNQ3* | ID | 602232 | R227Q | Missense | GOF | Moderate ID | Sands et al., 2019 |
| *KCNQ3* | EOEE | 602232 | V359L and D542N | Missense | LOF | Severe GDD | Ambrosino et al., 2018b |
| *KCNQ5* | ID and EP | 607357 | S448I | Missense | LOF | Severe ID | Lehman et al., 2017 |
| *KCNQ5* | ID and EP | 607357 | V145G | Missense | LOF | Mild ID | Lehman et al., 2017 |
| *KCNQ5* | ID and EP | 607357 | L341I | Missense | LOF | Moderate ID | Lehman et al., 2017 |
| *KCNQ5* | ID and EP | 607357 | P369R | Missense | GOF | Profound ID | Lehman et al., 2017 |
| *KCNQ5* | ID and EP | 607357 | V133* | Nonsense | LOF | Mild ID | Rosti et al., 2019 |
| **Name of the gene** | **Syndrome/Phenotype** | **OMIM number** | **Protein change** | **Type of mutation** | **Electrophysiological study result** | **Severity of the GDD/ID** | **Reference** |
| *KCNMA1* | ID, EP and cerebellar atrophy | 600150 | R458* | Nonsense | LOF | Severe ID | Yesil et al., 2018 |
| *KCNMA1* | ID, EP and ASD | 600150 | A138V | Missense | LOF | Severe ID | Laumonnier et al., 2006 |
| *KCNMA1* | ID | 600150 | S351Y | Missense | LOF | Mild ID | Liang et al., 2019 |
| *KCNMA1* | ID | 600150 | G356R | Missense | LOF | Borderline ID | Liang et al., 2019 |
| *KCNMA1* | ID | 600150 | G375R | Missense | LOF | Severe ID | Liang et al., 2019 |
| *KCNMA1* | ID | 600150 | G375R | Missense | LOF | Severe ID | Liang et al., 2019 |
| *KCNMA1* | ID. | 600150 | G375R | Missense | LOF | Severe ID | Liang et al., 2019 |
| *KCNMA1* | ID | 600150 | N449fs and  C413Y | Missense | LOF | ID | Liang et al., 2019 |
| *KCNMA1* | ID | 600150 | I663V | Missense | LOF | ID | Liang et al., 2019 |
| *KCNMA1* | ID | 600150 | P805L | Missense | LOF | ID | Liang et al., 2019 |
| *KCNMA1* | ID | 600150 | D984N | Missense | LOF | ID | Liang et al., 2019 |
| *KCNMA1* | GDD and paroxysmal non-kinesigenic dyskinesia | 600150 | E884K | Missense | Unknown | GDD | Zhang Z.B. et al., 2015 |
| *KCNMA1* | GDD and paroxysmal non-kinesigenic dyskinesia | 600150 | N1053S | Missense | Unknown | GDD | Zhang Z.B. et al., 2015 |
| *KCNMA1* | GDD, EP and severe cerebellar atrophy | 600150 | Y676Lfs∗7 | Nonsense | LOF | GDD | Tabarki et al., 2016 |
| *KCNMA1* | GDD, EP and severe cerebellar atrophy | 600150 | Y676Lfs∗7 | Nonsense | LOF | GDD | Tabarki et al., 2016 |
| *KCNC1* | ID | 176258 | R339* | Nonsense | LOF | Severe ID | Poirier et al., 2017 |
| *KCNC1* | ID | 176258 | R339* | Nonsense | LOF | Moderate ID | Poirier et al., 2017 |
| *KCNC1* | ID | 176258 | R339* | Nonsense | LOF | Severe ID | Poirier et al., 2017 |
| *KCNC1* | ID | 176258 | T399M | Missense | LOF | Moderate ID | Park et al., 2019 |
| *KCNC1* | ID and EP | 176258 | A421V | Missense | LOF | Severe ID | Cameron et al., 2019 |
| *KCNC1* | ID and EP | 176258 | A421V | Missense | LOF | Severe ID | Cameron et al., 2019 |
| *KCNC1* | ID and EP | 176258 | A421V | Missense | LOF | Moderate-severe ID | Cameron et al., 2019 |
| *KCNC1* | ID and EP | 176258 | A421V | Missense | LOF | Moderate-severe ID | Cameron et al., 2019 |
| **Name of the gene** | **Syndrome/Phenotype** | **OMIM number** | **Protein change** | **Type of mutation** | **Electrophysiological study result** | **Severity of the GDD/ID** | **Reference** |
| *KCNC1* | ID and EP | 176258 | A421V | Missense | LOF | GDD | Cameron et al., 2019 |
| *KCNC1* | ID and EP | 176258 | A421V | Missense | LOF | Moderate ID | Cameron et al., 2019 |
| *KCNC1* | ID and ASD | 176258 | R317H | Missense | LOF | Mild ID | Cameron et al., 2019 |
| *KCNC1* | ID | 176258 | Q492X | Missense | LOF | Moderate ID | Cameron et al., 2019 |
| *KCNC1* | ID | 176258 | Q492X | Missense | LOF | Moderate ID | Cameron et al., 2019 |
| *KCNJ6* | Keppen–Lubinsky Syndrome | 600877 | T152del | Deletion | LOF | Severe ID | Masotti et al., 2015 |
| *KCNJ6* | Keppen–Lubinsky Syndrome | 600877 | T152del | Deletion | LOF | Severe ID | Masotti et al., 2015 |
| *KCNJ6* | Keppen–Lubinsky Syndrome | 600877 | G154S | Missense | Unknown | Severe ID | Masotti et al., 2015 |
| *KCNB1* | Epileptic encephalopathy | 600397 | S347R | Missense | LOF | Severe ID | Torkamani et al., 2014 |
| *KCNB1* | Epileptic encephalopathy | 600397 | G379R | Missense | LOF | Severe ID | Torkamani et al., 2014 |
| *KCNB1* | Epileptic encephalopathy | 600397 | T374I | Missense | LOF | Severe ID | Torkamani et al., 2014 |
| *KCNB1* | Atypical Rett syndrome | 600397 | G379R | Missense | LOF | Severe ID | Srivastava et al., 2018 |
| *KCNB1* | Atypical Rett syndrome | 600397 | T374I | Missense | LOF | Severe ID | Srivastava et al., 2018 |
| *KCNB1* | West syndrome. | 600397 | W370* | Nonsense | LOF | Severe ID | Krey et al., 2019 |
| *KCNB1* | GDD, ADHD, and  ASD | 600397 | R583* | Nonsense | LOF | Severe GDD | de Kovel et al., 2017 |
| *KCNB1* | West syndrome | 600397 | K502fs | Frameshift | LOF | Severe GDD | de Kovel et al., 2017 |
| *KCNB1* | GDD, ADHD, and  ASD | 600397 | F416L | Missense | Unknown | Severe GDD | de Kovel et al., 2017 |
| *KCNB1* | GDD and EP | 600397 | F416L | Missense | Unknown | Severe GDD | de Kovel et al., 2017 |
| *KCNB1* | GDD and EP | 600397 | G401R | Missense | LOF | Severe GDD | de Kovel et al., 2017 |
| *KCNB1* | GDD and EP | 600397 | C397Y | Missense | Unknown | Severe GDD | de Kovel et al., 2017 |
| *KCNB1* | GDD and EP | 600397 | K391N | Missense | Unknown | Severe GDD | de Kovel et al., 2017 |
| *KCNB1* | West syndrome and ASD | 600397 | P385T | Missense | Unknown | Severe GDD | de Kovel et al., 2017 |
| *KCNB1* | GDD | 600397 | G381R | Missense | Unknown | Severe GDD | de Kovel et al., 2017 |
| **Name of the gene** | **Syndrome/Phenotype** | **OMIM number** | **Protein change** | **Type of mutation** | **Electrophysiological study result** | **Severity of the GDD/ID** | **Reference** |
| *KCNB1* | GDD, EP, and ASD | 600397 | G379R | Missense | LOF | Severe GDD | de Kovel et al., 2017 |
| *KCNB1* | GDD, EP | 600397 | V378A | Missense | LOF | Severe GDD | de Kovel et al., 2017 |
| *KCNB1* | GDD, EP and ASD | 600397 | T374I | Missense | LOF | Severe GDD | de Kovel et al., 2017 |
| *KCNB1* | GDD, EP, ADHD and  ASD | 600397 | W369* | Nonsense | LOF | Severe GDD | de Kovel et al., 2017 |
| *KCNB1* | GDD and EP | 600397 | S363fs*13 | Nonsense | LOF | Moderate GDD | de Kovel et al., 2017 |
| *KCNB1* | GDD and EP | 600397 | S347R | Missense | LOF | Severe GDD | de Kovel et al., 2017 |
| *KCNB1* | West syndrome | 600397 | R312H | Missense | Unknown | Severe GDD | de Kovel et al., 2017 |
| *KCNB1* | GDD, EP, and ASD | 600397 | R312H | Missense | Unknown | Severe GDD | de Kovel et al., 2017 |
| *KCNB1* | GDD, EP, and ASD | 600397 | R306C | Missense | LOF | Severe GDD | de Kovel et al., 2017 |
| *KCNB1* | GDD and ADHD | 600397 | R306C | Missense | LOF | Severe GDD | de Kovel et al., 2017 |
| *KCNB1* | GDD, EP and ADHD | 600397 | Q228* | Nonsense | LOF | Severe GDD | de Kovel et al., 2017 |
| *KCNB1* | GDD and EP | 600397 | L211P | Missense | Unknown | Moderate GDD | de Kovel et al., 2017 |
| *KCNB1* | GDD and EP | 600397 | T210K | Missense | Unknown | Moderate GDD | de Kovel et al., 2017 |
| *KCNB1* | ID, EP and ASD | 600397 | I199F | Missense | LOF | ID | Calhoun et al., 2017 |
| *KCNC3* | ID and ataxia | 176264 | D129N | Missense | GOF | Severe ID | Duarri et al., 2015 |
| *KCNC3* | ID | 176264 | V535M | Missense | GOF | Mild ID | Duarri et al., 2015 |
| *KCNC3* | ID and ataxia | 176264 | R423H | Missense | LOF | Mild ID | Duarri et al., 2015 |
| *KCNT1* | EP and ID | 608167 | R428Q | Missense | GOF | Severe ID | Alsaleem et al., 2019 |
| *KCNT1* | EIEE and severe dystonia | 608167 | L437F | Missense | GOF | Severe ID | Gertler et al., 2019 |
| *KCNT1* | EIMFS | 608167 | R428Q | Missense | GOF | Severe ID | Bearden et al., 2014 |
| *KCNT1* | EIMFS | 608167 | R933G | Missense | Unknown | Severe ID | Zhang et al., 2017 |
| *KCNT1* | EIMFS | 608167 | K629E | Missense | Unknown | Severe ID | Zhang et al., 2017 |
| *KCNT1* | EIMFS | 608167 | V271F | Missense | GOF | Severe ID | Zhang et al., 2017 |
| **Name of the gene** | **Syndrome/Phenotype** | **OMIM number** | **Protein change** | **Type of mutation** | **Electrophysiological study result** | **Severity of the GDD/ID** | **Reference** |
| *KCNT1* | EIMFS | 608167 | R428Q | Missense | GOF | Severe ID | Zhang et al., 2017 |
| *KCNT1* | EIMFS | 608167 | A934T | Missense | GOF | Severe ID | Zhang et al., 2017 |
| *KCNT1* | EIMFS | 608167 | R474H | Missense | GOF | Severe ID | Zhang et al., 2017 |
| *KCNT1* | EIMFS | 608167 | R1114W | Missense | GOF | Severe ID | Numis et al., 2018 |
| *KCNT1* | EIMFS | 608167 | A259D | Missense | GOF | Severe ID | Numis et al., 2018 |
| *KCNT1* | EIMFS | 608167 | M516V | Missense | GOF | Severe ID | Numis et al., 2018 |
| *KCNT1* | EIMFS | 608167 | R428Q | Missense | GOF | Severe ID | Numis et al., 2018 |
| *KCNT1* | EIMFS | 608167 | R428Q | Missense | GOF | Profound GDD | Barcia et al., 2019 |
| *KCNT1* | EIMFS | 608167 | L437F | Missense | GOF | Profound GDD | Barcia et al., 2019 |
| *KCNT1* | EIMFS | 608167 | R474C | Missense | GOF | Profound GDD | Barcia et al., 2019 |
| *KCNT1* | EIMFS | 608167 | R474H | Missense | GOF | Profound GDD | Barcia et al., 2019 |
| *KCNT1* | EIMFS | 608167 | R474H | Missense | GOF | Profound GDD | Barcia et al., 2019 |
| *KCNT1* | EIMFS | 608167 | M516V | Missense | GOF | Profound GDD | Barcia et al., 2019 |
| *KCNT1* | EIMFS | 608167 | M896I | Missense | GOF | Profound ID | Barcia et al., 2019 |
| *KCNT1* | EIMFS-West syndrome | 608167 | A934T | Missense | GOF | Profound ID | Barcia et al., 2019 |
| *KCNT1* | EIMFS | 608167 | A934T | Missense | GOF | Profound ID | Barcia et al., 2019 |
| *KCNT1* | EIMFS | 608167 | A965V | Missense | GOF | Profound DD | Barcia et al., 2019 |
| *KCNT1* | EIMFS-West syndrome | 608167 | R1106P | Missense | GOF | Profound DD | Barcia et al., 2019 |
| *KCNT1* | Ohtahara syndrome | 608167 | A966T | Missense | GOF | Profound DD | Martin et al., 2014 |
| *KCNT1* | EIMFS or Ohtahara syndrome | 608167 | R474C | Missense | GOF | Profound GDD | Ohba et al., 2015 |
| *KCNT1* | EIMFS | 608167 | Q270E | Missense | Unknown | Profound GDD | Ohba et al., 2015 |
| *KCNT1* | EIMFS | 608167 | P409S | Missense | Unknown | Profound GDD | Ohba et al., 2015 |
| *KCNT1* | EIMFS | 608167 | G288S | Missense | Unknown | Profound GDD | Ohba et al., 2015 |
| *KCNT1* | EIMFS | 608167 | R474H | Missense | GOF | Profound GDD | Ohba et al., 2015 |
| **Name of the gene** | **Syndrome/Phenotype** | **OMIM number** | **Protein change** | **Type of mutation** | **Electrophysiological study result** | **Severity of the GDD/ID** | **Reference** |
| *KCNT1* | EIMFS | 608167 | A934T | Missense | GOF | Profound GDD | Ohba et al., 2015 |
| *KCNT1* | West syndrome | 608167 | R474H | Missense | GOF | Profound GDD | Ohba et al., 2015 |
| *KCNT1* | EIMFS | 608167 | R474H | Missense | GOF | Profound GDD | Ohba et al., 2015 |
| *KCNT1* | EIMFS | 608167 | P924L | Missense | Unknown | Profound GDD | Ohba et al., 2015 |
| *KCNT1* | EIMFS | 608167 | A477T | Missense | Unknown | Profound GDD | Ohba et al., 2015 |
| *KCNT1* | EIMFS | 608167 | R428Q | Missense | GOF | Profound GDD | Ohba et al., 2015 |
| *KCNT1* | ID, EP and cerebellar ataxia | 608167 | R565H | Missense | Unknown | Severe ID | Hansen et al., 2017 |
| *KCNT1* | ID, EP and cerebellar ataxia | 608167 | R565H | Missense | Unknown | Severe ID | Hansen et al., 2017 |
| *KCNT1* | EIMFS | 608167 | R474C | Missense | Unknown | Severe GDD | Shimada et al., 2014 |
| *KCNT1* | Leukoencephalopathy, ID and severe myoclonic EP | 608167 | F932I | Missense | Unknown | Severe ID | Vanderver et al., 2014 |
| *KCNT1* | ID, EP, delayed myelination and leukoencephalopathy | 608167 | F932I | Missense | LOF | Severe GDD | Evely KM et al., 2017 |
| *KCNT1* | EIMFS | 608167 | R428Q | Missense | GOF | Severe GDD | Borlot F et al., 2020 |
| *KCNT1* | EIMFS | 608167 | D480N | Missense | Unknown | Severe GDD | Borlot F et al., 2020 |
| *KCNT1* | EIMFS/West syndrome | 608167 | R950Q | Missense | GOF | Severe GDD | Borlot F et al., 2020 |
| *KCNT1* | EIMFS/West syndrome | 608167 | A934T | Missense | Unknown | Severe GDD | Borlot F et al., 2020 |
| *KCNT1* | EIMFS | 608167 | K629E | Missense | Unknown | Severe GDD | Borlot F et al., 2020 |
| *KCNT1* | EIMFS | 608167 | R474H | Missense | GOF | Severe GDD | Borlot F et al., 2020 |
| *KCNT1* | EIMFS | 608167 | G288S | Missense | Unknown | Severe GDD | Borlot F et al., 2020 |
| *KCNT1* | EIMFS | 608167 | A934T | Missense | GOF | Severe GDD | Borlot F et al., 2020 |
| *KCNT1* | ID | 608167 | G288S | Missense | Unknown | Severe GDD | Borlot F et al., 2020 |
| *KCNT1* | West syndrome | 608167 | G288S | Missense | Unknown | Severe GDD | Borlot F et al., 2020 |
| *KCNT1* | EIMFS | 608167 | G288S | Missense | Unknown | Severe GDD | Borlot F et al., 2020 |
| *KCNT1* | Unclassified EOEE | 608167 | C377S | Missense | Unknown | Severe GDD | Borlot F et al., 2020 |
| **Name of the gene** | **Syndrome/Phenotype** | **OMIM number** | **Protein change** | **Type of mutation** | **Electrophysiological study result** | **Severity of the GDD/ID** | **Reference** |
| *KCNT1* | EIMFS | 608167 | R474C | Missense | GOF | Severe GDD | Borlot F et al., 2020 |
| *KCNT1* | EIMFS | 608167 | L962P | Missense | Unknown | Severe GDD | Borlot F et al., 2020 |
| *KCNT1* | EIMFS | 608167 | R950Q | Missense | GOF | Severe GDD | Borlot F et al., 2020 |
| *KCNT1* | EIMFS | 608167 | A934T | Missense | GOF | Severe GDD | Borlot F et al., 2020 |
| *KCNT1* | ID and EP | 608167 | R474H | Missense | GOF | Severe GDD | Borlot F et al., 2020 |
| *KCNT1* | Unclassified EOEE | 608167 | R85S | Missense | GOF | Severe GDD | Borlot F et al., 2020 |
| *KCNT1* | EIMFS | 608167 | L274I | Missense | GOF | Severe GDD | Borlot F et al., 2020 |
| *KCNT1* | EIMFS | 608167 | R474H | Missense | GOF | Severe GDD | Borlot F et al., 2020 |
| *KCNT1* | Unclassified EOEE | 608167 | R961H | Missense | Unknown | Severe GDD | Borlot F et al., 2020 |
| *KCNT1* | Unclassified EOEE | 608167 | R474C | Missense | GOF | Severe GDD | Borlot F et al., 2020 |
| *KCNT1* | EIMFS | 608167 | R428Q | Missense | GOF | Severe GDD | Borlot F et al., 2020 |
| *KCNT1* | Unclassified EOEE | 608167 | A934T | Missense | GOF | Severe GDD | Borlot F et al., 2020 |
| *KCNT1* | EIMFS | 608167 | Q270E | Missense | Unknown | Severe GDD | Borlot F et al., 2020 |
| *KCNT1* | ID | 608167 | R398Q | Missense | Unknown | Severe GDD | Borlot F et al., 2020 |
| *KCNT1* | EIMFS | 608167 | A934T | Missense | GOF | Severe GDD | Borlot F et al., 2020 |
| *KCNT1* | EIMFS | 608167 | G288S | Missense | Unknown | Profound GDD | Rizzo F et al., 2016 |
| *KCNT1* | EIMFS | 608167 | M516V | Missense | GOF | Profound GDD | Rizzo F et al., 2016 |
| *KCNT1* | EIMFS | 608167 | C377S | Missense | Unknown | Profound GDD | Kawasaki Y et al., 2017 |
| *KCNT1* | EIMFS | 608167 | R474C | Missense | GOF | Profound GDD | Kawasaki Y et al., 2017 |
| *KCNT1* | EIMFS | 608167 | R474H | Missense | GOF | Profound GDD | Kawasaki Y et al., 2017 |
| *KCNT1* | EIMFS | 608167 | V271F | Missense | GOF | Profound GDD | McTague A et al., 2018 |
| *KCNT1* | EIMFS | 608167 | L274I | Missense | GOF | Profound GDD | McTague A et al., 2018 |
| **Name of the gene** | **Syndrome/Phenotype** | **OMIM number** | **Protein change** | **Type of mutation** | **Electrophysiological study result** | **Severity of the GDD/ID** | **Reference** |
| *KCNT1* | EIMFS | 608167 | M896K | Missense | Unknown | Profound GDD | McTague A et al., 2018 |
| *KCNT1* | EIMFS | 608167 | R950Q | Missense | GOF | Severe GDD | McTague A et al., 2018 |
| *KCNT1* | EIMFS | 608167 | A934T | Missense | GOF | Profound GDD | McTague A et al., 2018 |
| *KCNT1* | EIMFS | 608167 | A934T | Missense | GOF | Profound GDD | McTague A et al., 2018 |
| *KCNT1* | EIMFS | 608167 | A934T | Missense | GOF | Profound GDD | McTague A et al., 2018 |
| *KCNT1* | EIMFS | 608167 | A934T | Missense | GOF | Profound GDD | McTague A et al., 2018 |
| *KCNT1* | EIMFS | 608167 | A934T | Missense | GOF | Profound GDD | McTague A et al., 2018 |
| *KCNT1* | EIMFS | 608167 | R950Q | Missense | GOF | Severe GDD | Dilena R et. Al., 2018 |
| *KCNT1* | EIMFS | 608167 | E893K | Missense | GOF | Severe GDD | Dilena R et. Al., 2018 |
| *KCNT2* | EOEE | 610044 | F240L | Missense | LOF | Severe GDD | Gururaj S et al., 2017 |
| *KCNT2* | West syndrome then Lennox-Gastaut syndrome | 610044 | R190H | Missense | GOF | Severe GDD | Ambrosino P et al., 2018a |
| *KCNT2* | EIMFS | 610044 | R190P | Missense | GOF | Severe GDD | Ambrosino P et al.,2018a |
| *KCNT2* | EIMFS-like EOEE | 610044 | L48Qfs43* | Nonsense | LOF | Severe GDD | Mao X et al., 2020 |
| *KCNT2* | EIMFS | 610044 | K564* | Nonsense | LOF | Severe GDD | Mao X et al., 2020 |
| *KCTD3* | ID and EP | 613272 | P346Tfs*4 | Nonsense | LOF | Severe GDD | Faqeih et al., 2018 |
| *KCTD3* | ID and EP | 613272 | P346Tfs*4 | Nonsense | LOF | Severe GDD | Faqeih et al., 2018 |
| *KCTD3* | ID and EP | 613272 | P346Tfs*4 | Nonsense | LOF | Severe GDD | Faqeih et al., 2018 |
| *KCTD3* | ID and EP | 613272 | P346Tfs*4 | Nonsense | LOF | Severe GDD | Faqeih et al., 2018 |
| *KCTD3* | ID and EP | 613272 | R56* | Nonsense | LOF | Severe GDD | Faqeih et al., 2018 |
| *KCTD3* | ID and EP | 613272 | R56* | Nonsense | LOF | Severe GDD | Faqeih et al., 2018 |
| *KCTD3* | ID and EP | 613272 | P346Tfs*4 | Nonsense | LOF | Moderate GDD | Faqeih et al., 2018 |
| *KCTD3* | ID and EP | 613272 | P346Tfs*4 | Nonsense | LOF | Severe ID | Alazami et al., 2015 |
| *KCTD3* | ID and EP | 613272 | P346Tfs*4 | Nonsense | LOF | Severe ID | Trujillano et al., 2017 |
| **Name of the gene** | **Syndrome/Phenotype** | **OMIM number** | **Protein change** | **Type of mutation** | **Electrophysiological study result** | **Severity of the GDD/ID** | **Reference** |
| *KCNJ11* | DEND | 600937 | Q52R | Missense | GOF | GDD | Flanagan et al., 2006 |
| *KCNJ11* | DEND | 600937 | G53D | Missense | GOF | GDD | Flanagan et al., 2006 |
| *KCNJ11* | DEND | 600937 | V59G | Missense | GOF | GDD | Flanagan et al., 2006 |
| *KCNJ11* | DEND | 600937 | C166Y | Missense | GOF | Profound GDD | Flanagan et al., 2006 |
| *KCNJ11* | DEND | 600937 | I296L | Missense | GOF | GDD | Flanagan et al., 2006; Proks et al., 2005 |
| *KCNJ11* | I-DEND | 600937 | R201C | Missense | GOF | GDD | Flanagan et al., 2006 |
| *KCNJ11* | I-DEND | 600937 | R201C | Missense | GOF | GDD | Flanagan et al., 2006 |
| *KCNJ11* | I-DEND | 600937 | V59M | Missense | GOF | GDD | Flanagan et al., 2006 |
| *KCNJ11* | I-DEND | 600937 | V59M | Missense | GOF | GDD | Flanagan et al., 2006 |
| *KCNJ11* | I-DEND | 600937 | V59M | Missense | GOF | Moderate ID | Flanagan et al., 2006 |
| *KCNJ11* | I-DEND | 600937 | V59M | Missense | GOF | Moderate ID | Flanagan et al., 2006 |
| *KCNJ11* | I-DEND | 600937 | H46L | Missense | GOF | Moderate ID | Fendler et al., 2013 |
| *KCNJ11* | I-DEND | 600937 | G53D | Missense | GOF | Moderate ID | Fendler et al., 2013 |
| *KCNJ11* | I-DEND | 600937 | G53D | Missense | GOF | Moderate ID | Fendler et al., 2013 |
| *KCNJ11* | I-DEND | 600937 | V59M | Missense | GOF | Moderate ID | Fendler et al., 2013 |
| *KCNJ11* | I-DEND | 600937 | V59M | Missense | GOF | Moderate ID | Shah et al., 2012 |
| *KCNJ11* | I-DEND | 600937 | V59M | Missense | GOF | Moderate ID | Shah et al., 2012 |
| *KCNJ11* | I-DEND | 600937 | V59M | Missense | GOF | Moderate ID | Shah et al., 2012 |
| *KCNJ11* | I-DEND | 600937 | V59M | Missense | GOF | Moderate ID | Shah et al., 2012 |
| *KCNJ11* | I-DEND | 600937 | V59M | Missense | GOF | Moderate ID | Shah et al., 2012 |
| *KCNJ11* | I-DEND | 600937 | V59M | Missense | GOF | Moderate ID | Shah et al., 2012 |
| *KCNJ11* | I-DEND | 600937 | V59M | Missense | GOF | Moderate ID | Shah et al., 2012 |
| *KCNJ11* | I-DEND | 600937 | V59M | Missense | GOF | Moderate ID | Carmody et al., 2016 |
| **Name of the gene** | **Syndrome/Phenotype** | **OMIM number** | **Protein change** | **Type of mutation** | **Electrophysiological study result** | **Severity of the GDD/ID** | **Reference** |
| *KCNJ11* | I-DEND | 600937 | V59M | Missense | GOF | Moderate ID | Carmody et al., 2016 |
| *KCNJ11* | I-DEND | 600937 | V59M | Missense | GOF | Moderate ID | Carmody et al., 2016 |
| *KCNJ11* | I-DEND | 600937 | V59M | Missense | GOF | Moderate ID | Carmody et al., 2016 |
| *KCNJ11* | I-DEND | 600937 | V59M | Missense | GOF | Moderate ID | Carmody et al., 2016 |
| *KCNJ11* | I-DEND | 600937 | V59M | Missense | GOF | Moderate ID | Carmody et al., 2016 |
| *KCNJ11* | I-DEND | 600937 | V59M | Missense | GOF | Moderate ID | Carmody et al., 2016 |
| *KCNJ11* | I-DEND | 600937 | V59M | Missense | GOF | Moderate ID | Carmody et al., 2016 |
| *KCNJ11* | I-DEND | 600937 | V59A | Missense | GOF | Moderate ID | Carmody et al., 2016 |
| *KCNJ11* | I-DEND | 600937 | Y330C | Missense | GOF | Moderate ID | Carmody et al., 2016 |
| *KCNJ11* | I-DEND | 600937 | V59M | Missense | GOF | Moderate ID | Mohamadi et al., 2010 |
| *KCNJ11* | I-DEND | 600937 | homS225T, del | Missense and deletion | GOF and LOF | Moderate ID | Lin et al., 2013 |
| *KCNJ11* | I-DEND | 600937 | V59M | Missense | GOF | Moderate ID | Massa et al., 2005 |
| *KCNJ11* | I-DEND | 600937 | V59M | Missense | GOF | Moderate ID | Massa et al., 2005 |
| *KCNJ11* | I-DEND | 600937 | K170N | Missense | GOF | Moderate ID | Massa et al., 2005 |

**Abbreviations: ADHD; attention deficit hyperactive disorder, ASD; autism spectrum disorder, DEND: developmental delay, epilepsy, and neonatal diabetes, EEG; electroencephalograph, EP; epilepsy, EIMFS; epilepsy of infancy with migrating focal seizures, EAST: epilepsy, ataxia, sensorineural deafness, and tubulopathy, FHEIG: facial dysmorphism, hypertrichosis, epilepsy, intellectual disability/developmental delay, and gingival overgrowth, GDD; global developmental delay, GOF: gain of function, ID: intellectual disability, I-DEND: neonatal diabetes with moderate developmental delay and/or muscle weakness but not epilepsy, LOF: loss of function, MRI; magnetic resonance imaging, SeSAME: seizures, sensorineural deafness, ataxia, mental retardation, and electrolyte imbalance, TBS: Temple–Baraitser Syndrome, ZLS; Zimmermann–Laband syndrome.**

**References**

Abidi, A., Devaux, J.J., Molinari, F., Alcaraz, G., Michon, F.X., Sutera-Sardo, J., et al. (2015). A recurrent KCNQ2 pore mutation causing early onset epileptic encephalopathy has a moderate effect on M current but alters subcellular localization of Kv7 channels. *Neurobiol Dis*. 80, 80-92. doi: 10.1016/j.nbd.2015.04.017. Epub 2015 May 22.

Ambrosino, P., Soldovieri, M.V., Bast, T., Turnpenny. P.D., Uhrig. S., Biskup. S, et al. (2018a). De novo gain-of-function variants in KCNT2 as a novel cause of developmental and epileptic encephalopathy. *Ann Neurol*. 83, 1198-1204. doi: 10.1002/ana.25248.

Ambrosino, P., Freri, E., Castellotti, B., Soldovieri, M.V., Mosca. I., Manocchio, L., et al (2018b). Kv7.3 Compound Heterozygous Variants in Early Onset Encephalopathy Reveal Additive Contribution of C-Terminal Residues to PIP2-Dependent K+ Channel Gating. *Mol Neurobiol*. 55, 7009-7024. doi: 10.1007/s12035-018-0883-5. Epub 2018 Jan 30.

Al Dhaibani, M. A., El-Hattab, A. W., Holroyd, K. B., Orthmann-Murphy, J., Larson, V. A., Siddiqui, K. A., et al. (2018). Novel mutation in the KCNJ10 gene in three siblings with seizures, ataxia and no electrolyte abnormalities. *J. Neurogenet.* 32, 1–5. doi:10.1080/01677063.2017.1404057.

Alazami, A. M., Patel, N., Shamseldin, H. E., Anazi, S., Al-Dosari, M. S., Alzahrani, F., et al. (2015). Accelerating novel candidate gene discovery in neurogenetic disorders via whole-exome sequencing of prescreened multiplex consanguineous families. *Cell Rep.* 10, 148–161. doi:10.1016/j.celrep.2014.12.015.

Alsaleem, M., Carrion, V., Weinstock, A., and Chandrasekharan, P. (2019). Infantile refractory seizures due to de novo KCNT 1 mutation. *BMJ Case Rep.* 12. doi:10.1136/bcr-2019-231178.

Barcia, G., Chemaly, N., Kuchenbuch, M., Eisermann, M., Gobin-Limballe, S., Ciorna, V., et al. (2019). Epilepsy with migrating focal seizures: KCNT1 mutation hotspots and phenotype variability. *Neurol. Genet.* 5, e363. doi:10.1212/NXG.0000000000000363.

Bauer, C. K., Calligari, P., Radio, F. C., Caputo, V., Dentici, M. L., Falah, N., et al. (2018). Mutations in KCNK4 that Affect Gating Cause a Recognizable Neurodevelopmental Syndrome. *Am. J. Hum. Genet.* 103, 621–630. doi:10.1016/j.ajhg.2018.09.001.

Bauer, C. K., Schneeberger, P. E., Kortum, F., Altmuller, J., Santos-Simarro, F., Baker, L., et al. (2019). Gain-of-Function Mutations in KCNN3 Encoding the Small-Conductance Ca(2+)-Activated K(+) Channel SK3 Cause Zimmermann-Laband Syndrome. *Am. J. Hum. Genet.* 104, 1139–1157. doi:10.1016/j.ajhg.2019.04.012.

Bearden, D., Strong, A., Ehnot, J., DiGiovine, M., Dlugos, D., and Goldberg, E. M. (2014). Targeted treatment of migrating partial seizures of infancy with quinidine. *Ann. Neurol.* 76, 457–461. doi:10.1002/ana.24229.

Borlot, F., Abushama, A., Morrison-Levy, N., Jain, P., Puthenveettil, Vinayan, K., et al.(2020). KCNT1-related epilepsy: An international multicenter cohort of 27 pediatric cases. *Epilepsia*. 61, 679-692. doi: 10.1111/epi.16480. Epub 2020 Mar 13.

Borgatti, R., Zucca, C., Cavallini, A., Ferrario, M., Panzeri, C., Castaldo, P., et al. (2004). A novel mutation in KCNQ2 associated with BFNC, drug resistant epilepsy, and mental retardation. *Neurology*. 63, 57–65. doi:10.1212/01.wnl.0000132979.08394.6d.

Calhoun, J.D., Vanoye, C.G., Kok. F., George, A.L .Jr., Kearney, J,A. (2017). Characterization of a KCNB1 variant associated with autism, intellectual disability, and epilepsy. *Neurol Genet*. 3, e198. doi: 10.1212/NXG.0000000000000198. eCollection 2017 Dec.

Cameron, J. M., Maljevic, S., Nair, U., Aung, Y. H., Cogne, B., Bezieau, S., et al. (2019). Encephalopathies with KCNC1 variants: genotype-phenotype-functional correlations. *Ann. Clin. Transl. Neurol.* 6, 1263–1272. doi:10.1002/acn3.50822.

Carmody, D., Pastore, A. N., Landmeier, K. A., Letourneau, L. R., Martin, R., Hwang, J. L., et al. (2016). Patients with KCNJ11-related diabetes frequently have neuropsychological impairments compared with sibling controls. *Diabet. Med.* 33, 1380–1386. doi:10.1111/dme.13159.

de Kovel, C. G. F., Syrbe, S., Brilstra, E. H., Verbeek, N., Kerr, B., Dubbs, H., et al. (2017). Neurodevelopmental Disorders Caused by De Novo Variants in KCNB1 Genotypes and Phenotypes. *JAMA Neurol.* 74, 1228–1236. doi:10.1001/jamaneurol.2017.1714.

Dilena, R., DiFrancesco, J.C., Soldovieri, M.V., Giacobbe, A., Ambrosino, P., Mosca, I., et al. (2018). Early Treatment with Quinidine in 2 Patients with Epilepsy of Infancy with Migrating Focal Seizures (EIMFS) Due to Gain-of-Function KCNT1 Mutations: Functional Studies, Clinical Responses, and Critical Issues for Personalized Therapy. *Neurotherapeutics*. 15, 1112-1126. doi: 10.1007/s13311-018-0657-9.

Dimassi, S., Labalme, A., Ville, D., Calender, A., Mignot, C., Boutry-Kryza, N., et al. (2016). Whole-exome sequencing improves the diagnosis yield in sporadic infantile spasm syndrome. *Clin. Genet.* 89, 198–204. doi:10.1111/cge.12636.

Duarri, A., Nibbeling, E. A. R., Fokkens, M. R., Meijer, M., Boerrigter, M., Verschuuren-Bemelmans, C. C., et al. (2015). Functional analysis helps to define KCNC3 mutational spectrum in Dutch ataxia cases. *PLoS One*. 10, e0116599. doi:10.1371/journal.pone.0116599.

Evely, KM., Pryce, K.D., Bhattacharjee, A. (2017). The Phe932Ile mutation in KCNT1 channels associated with severe epilepsy, delayed myelination and leukoencephalopathy produces a loss-of-function channel phenotype. *Neuroscience*. 351, 65-70. doi: 10.1016/j.neuroscience.2017.03.035. Epub 2017 Mar 31.

Faqeih, E. A., Almannai, M., Saleh, M. M., AlWadei, A. H., Samman, M. M., and Alkuraya, F. S. (2018). Phenotypic characterization of KCTD3-related developmental epileptic encephalopathy. *Clin. Genet.* 93, 1081–1086. doi:10.1111/cge.13227.

Fendler, W., Pietrzak, I., Brereton, M. F., Lahmann, C., Gadzicki, M., Bienkiewicz, M., et al. (2013). Switching to sulphonylureas in children with iDEND syndrome caused by KCNJ11 mutations results in improved cerebellar perfusion. *Diabetes Care*. 36, 2311–2316. doi:10.2337/dc12-2166.

Flanagan, S. E., Edghill, E. L., Gloyn, A. L., Ellard, S., and Hattersley, A. T. (2006). Mutations in KCNJ11, which encodes Kir6.2, are a common cause of diabetes diagnosed in the first 6 months of life, with the phenotype determined by genotype. *Diabetologia*. 49, 1190–1197. doi:10.1007/s00125-006-0246-z.

Gertler, T. S., Thompson, C. H., Vanoye, C. G., Millichap, J. J., and George, A. L. J. (2019). Functional consequences of a KCNT1 variant associated with status dystonicus and early-onset infantile encephalopathy. *Ann. Clin. Transl. Neurol.* 6, 1606–1615. doi:10.1002/acn3.50847.

Gururaj, S., Palmer, E. E., Sheehan, G.D,, Kandula. T., Macintosh, R., Ying, K., et al. (2017). A De Novo Mutation in the Sodium-Activated Potassium Channel KCNT2 Alters Ion Selectivity and Causes Epileptic Encephalopathy. *Cell Rep*. 21, 926-933. doi: 10.1016/j.celrep.2017.09.088.

Hansen, N., Widman, G., Hattingen, E., Elger, C. E., and Kunz, W. S. (2017). Mesial temporal lobe epilepsy associated with KCNT1 mutation. *Seizure*. 45, 181–183. doi:10.1016/j.seizure.2016.12.018.

Helbig, K. L., Hedrich, U. B. S., Shinde, D. N., Krey, I., Teichmann, A.-C., Hentschel, J., et al. (2016). A recurrent mutation in KCNA2 as a novel cause of hereditary spastic paraplegia and ataxia. *Ann. Neurol.* 80. doi:10.1002/ana.24762.

Hewson, S., Puka, K., and Mercimek-Mahmutoglu, S. (2017). Variable expressivity of a likely pathogenic variant in KCNQ2 in a three-generation pedigree presenting with intellectual disability with childhood onset seizures. *Am. J. Med. Genet. A* 173, 2226–2230. doi:10.1002/ajmg.a.38281.

Hundallah, K., Alenizi, A., AlHashem, A., and Tabarki, B. (2016). Severe early-onset epileptic encephalopathy due to mutations in the KCNA2 gene: Expansion of the genotypic and phenotypic spectrum. *Eur. J. Paediatr. Neurol.* 20, 657–660. doi:10.1016/j.ejpn.2016.03.011.

Kara, B., Ekici, B., Ipekci, B., Aslanger, A. K., and Scholl, U. (2013). KCNJ10 gene mutation in an 8-year-old boy with seizures. *Acta Neurol. Belg.* 113, 75–77. doi:10.1007/s13760-012-0113-2.

Kato, M., Yamagata, T., Kubota, M., Arai, H., Yamashita, S., Nakagawa, T., et al. (2013). Clinical spectrum of early onset epileptic encephalopathies caused by KCNQ2 mutation. *Epilepsia*. 54, 1282–1287. doi:10.1111/epi.12200.

Kawasaki,Y., Kuki, I., Ehara, E., Murakami, Y., Okazaki, S., Kawawaki, H., et al. (2017). Three Cases of KCNT1 Mutations: Malignant Migrating Partial Seizures in Infancy with Massive Systemic to Pulmonary Collateral Arteries. *J Pediatr*. 191, 270-274. doi: 10.1016/j.jpeds.2017.08.057. Epub 2017 Oct 5.

Kaya, N., Alsagob, M., D’Adamo, M. C., Al-Bakheet, A., Hasan, S., Muccioli, M., et al. (2016). KCNA4 deficiency leads to a syndrome of abnormal striatum, congenital cataract and intellectual disability. *J. Med. Genet.* 53, 786–792. doi:10.1136/jmedgenet-2015-103637.

Kortum, F., Caputo, V., Bauer, C. K., Stella, L., Ciolfi, A., Alawi, M., et al. (2015). Mutations in KCNH1 and ATP6V1B2 cause Zimmermann-Laband syndrome. *Nat. Genet.* 47, 661–667. doi:10.1038/ng.3282.

Krey, I., Krois-Neudenberger, J., Hentschel, J., Syrbe, S., Polster, T., Hanker, B., et al. (2019). Genotype-phenotype correlation on 45 individuals with West syndrome. *Eur. J. Paediatr. Neurol.* 25, 134-138. doi:10.1016/j.ejpn.2019.11.010.

Kurihara, M., Ishiura, H., Sasaki, T., Otsuka, J., Hayashi, T., Terao, Y., et al. (2018). Novel De Novo KCND3 Mutation in a Japanese Patient with Intellectual Disability, Cerebellar Ataxia, Myoclonus, and Dystonia. *Cerebellum*. 17, 237–242. doi:10.1007/s12311-017-0883.

Laumonnier, F., Roger, S., Guerin, P., Molinari, F., M’rad, R., Cahard, D., et al. (2006). Association of a functional deficit of the BKCa channel, a synaptic regulator of neuronal excitability, with autism and mental retardation. *Am. J. Psychiatry*. 163, 1622–1629. doi:10.1176/ajp.2006.163.9.1622.

Lehman, A., Thouta, S., Mancini, G. M. S., Naidu, S., van Slegtenhorst, M., McWalter, K., et al. (2017). Loss-of-Function and Gain-of-Function Mutations in KCNQ5 Cause Intellectual Disability or Epileptic Encephalopathy. *Am. J. Hum. Genet.* 101, 65–74. doi:10.1016/j.ajhg.2017.05.016.

Liang, L., Li, X., Moutton, S., Schrier Vergano, S. A., Cogne, B., Saint-Martin, A., et al. (2019). De novo loss-of-function KCNMA1 variants are associated with a new multiple malformation syndrome and a broad spectrum of developmental and neurological phenotypes. *Hum. Mol. Genet.* 28, 2937–2951. doi:10.1093/hmg/ddz117.

Lin, Y.W., Li, A., Grasso, V., Battaglia, D., Crino, A., Colombo, C., et al. (2013). Functional characterization of a novel KCNJ11 in frame mutation-deletion associated with infancy-onset diabetes and a mild form of intermediate DEND: a battle between K(ATP) gain of channel activity and loss of channel expression. *PLoS One*. 8, e63758. doi:10.1371/journal.pone.0063758.

Martin, H. C., Kim, G. E., Pagnamenta, A. T., Murakami, Y., Carvill, G. L., Meyer, E., et al. (2014). Clinical whole-genome sequencing in severe early-onset epilepsy reveals new genes and improves molecular diagnosis. *Hum. Mol. Genet.* 23, 3200–3211. doi:10.1093/hmg/ddu030.

Masotti, A., Uva, P., Davis-Keppen, L., Basel-Vanagaite, L., Cohen, L., Pisaneschi, E., et al. (2015). Keppen-Lubinsky syndrome is caused by mutations in the inwardly rectifying K+ channel encoded by KCNJ6. *Am. J. Hum. Genet.* 96, 295–300. doi:10.1016/j.ajhg.2014.12.011.

Massa, O., Iafusco, D., D’Amato, E., Gloyn, A. L., Hattersley, A. T., Pasquino, B., et al. (2005). KCNJ11 activating mutations in Italian patients with permanent neonatal diabetes. *Hum. Mutat.* 25, 22–27. doi:10.1002/humu.20124.

Mao, X., Bruneau, N., Gao, Q., Becq, H., Jia, Z1., Xi, H., et al. (2020). The Epilepsy of Infancy With Migrating Focal Seizures: Identification of de novo Mutations of the KCNT2 Gene That Exert Inhibitory Effects on the Corresponding Heteromeric KNa1.1/KNa1.2 Potassium Channel. *Front Cell Neurosci*. 14, 1. doi: 10.3389/fncel.2020.00001. eCollection 2020.

McTague, A., Nair, U., Malhotra, S., Meyer, E., Trump, N., Gazina, E.V., et al. (2018). Clinical and molecular characterization of KCNT1-related severe early-onset epilepsy. *Neurology*. 90:e55-e66. doi: 10.1212/WNL.0000000000004762. Epub 2017 Dec 1.

Megarbane, A., Al-Ali, R., Choucair, N., Lek, M., Wang, E., Ladjimi, M., et al. (2016). Temple-Baraitser Syndrome and Zimmermann-Laband Syndrome: one clinical entity? *BMC Med. Genet.* 17, 42. doi:10.1186/s12881-016-0304-4.

Miceli, F., Striano, P., Soldovieri, M. V., Fontana, A., Nardello, R., Robbiano, A., et al. (2015b). A novel KCNQ3 mutation in familial epilepsy with focal seizures and intellectual disability. *Epilepsia*. 56, e15-20. doi:10.1111/epi.12887.

Mohamadi, A., Clark, L. M., Lipkin, P. H., Mahone, E. M., Wodka, E. L., and Plotnick, L. P. (2010). Medical and developmental impact of transition from subcutaneous insulin to oral glyburide in a 15-yr-old boy with neonatal diabetes mellitus and intermediate DEND syndrome: extending the age of KCNJ11 mutation testing in neonatal DM. *Pediatr.*

Numis, A.L., Nair, U., Datta, A.N., Sands, T.T., Oldham, M.S., Patel, A., et al (2018). Lack of response to quinidine in KCNT1-related neonatal epilepsy. *Epilepsia.* 59, 1889-1898. doi: 10.1111/epi.14551. Epub 2018 Sep 4.

Ohba, C., Kato, M., Takahashi, N., Osaka, H., Shiihara, T., Tohyama, J., et al. (2015). De novo KCNT1 mutations in early-onset epileptic encephalopathy. *Epilepsia*. 56, e121-8. doi:10.1111/epi.13072.

Papavasiliou, A., Foska, K., Ioannou, J., and Nagel, M. (2017). Epilepsy, ataxia, sensorineural deafness, tubulopathy syndrome in a European child with KCNJ10 mutations: A case report. *SAGE open Med. case reports*. 5, 2050313X17723549. doi:10.1177/2050313X17723549.

Poirier, K., Viot, G., Lombardi, L., Jauny, C., Billuart, P., and Bienvenu, T. (2017). Loss of Function of KCNC1 is associated with intellectual disability without seizures. *Eur. J. Hum. Genet.* 25, 560–564. doi:10.1038/ejhg.2017.3.

Proks, P., Girard, C., Haider, S., Gloyn, A. L., Hattersley, A. T., Sansom, M. S. P., et al. (2005). A gating mutation at the internal mouth of the Kir6.2 pore is associated with DEND syndrome. *EMBO Rep.* 6, 470–475. doi:10.1038/sj.embor.7400393.

Rizzo, F., Ambrosino, P., Guacci, A., Chetta, M., Marchese, G., Rocco, T., et al. Characterization of two de novoKCNT1 mutations in children with malignant migrating partial seizures in infancy. (2016). *Mol Cell Neurosci*. 72, 54-63. doi: 10.1016/j.mcn.2016.01.004. Epub 2016 Jan 16.

Sands, T. T., Miceli, F., Lesca, G., Beck, A. E., Sadleir, L. G., Arrington, D. K., et al. (2019). Autism and developmental disability caused by KCNQ3 gain-of-function variants. *Ann. Neurol.* 86, 181–192. doi:10.1002/ana.25522.

Scholl, U. I., Choi, M., Liu, T., Ramaekers, V. T., Hausler, M. G., Grimmer, J., et al. (2009). Seizures, sensorineural deafness, ataxia, mental retardation, and electrolyte imbalance (SeSAME syndrome) caused by mutations in KCNJ10. *Proc. Natl. Acad. Sci. U. S. A.* 106, 5842–5847. doi:10.1073/pnas.0901749106.

Severino, M., Lualdi, S., Fiorillo, C., Striano, P., De Toni, T., Peluso, S., et al. (2018). Unusual white matter involvement in EAST syndrome associated with novel KCNJ10 mutations. *J. Neurol.* 265, 1419–1425. doi:10.1007/s00415-018-8826-7.

Shah, R. P., Spruyt, K., Kragie, B. C., Greeley, S. A. W., and Msall, M. E. (2012). Visuomotor performance in KCNJ11-related neonatal diabetes is impaired in children with DEND-associated mutations and may be improved by early treatment with sulfonylureas. *Diabetes Care*. 35, 2086–2088. doi:10.2337/dc11-2225.

Shimada, S., Hirano, Y., Ito, S., Oguni, H., Nagata, S., Shimojima, K., et al. (2014). A novel KCNT1 mutation in a Japanese patient with epilepsy of infancy with migrating focal seizures. *Hum. genome Var.* 1, 14027. doi:10.1038/hgv.2014.27.

Sicca, F., Imbrici, P., D’Adamo, M. C., Moro, F., Bonatti, F., Brovedani, P., et al. (2011). Autism with seizures and intellectual disability: possible causative role of gain-of-function of the inwardly-rectifying K+ channel Kir4.1. *Neurobiol. Dis.* 43, 239–247. doi:10.1016/j.nbd.2011.03.016.

Simons, C., Rash, L. D., Crawford, J., Ma, L., Cristofori-Armstrong, B., Miller, D., et al. (2015). Mutations in the voltage-gated potassium channel gene KCNH1 cause Temple-Baraitser syndrome and epilepsy. *Nat. Genet.* 47, 73–77. doi:10.1038/ng.3153.

Smets, K., Duarri, A., Deconinck, T., Ceulemans, B., van de Warrenburg, B. P., Zuchner, S., et al. (2015). First de novo KCND3 mutation causes severe Kv4.3 channel dysfunction leading to early onset cerebellar ataxia, intellectual disability, oral apraxia and epilepsy. *BMC Med. Genet.* 16, 51. doi:10.1186/s12881-015-0200-3.

Soldovieri., M.V., Ambrosino, P., Mosca, I., De Maria, M., Moretto, E., Miceli, F., et al. (2016). Early-onset epileptic encephalopathy caused by a reduced sensitivity of Kv7.2 potassium channels to phosphatidylinositol 4,5-bisphosphate. *Sci Rep*. 6, 38167. doi: 10.1038/srep38167.

Soldovieri, M.V., Ambrosino, P., Mosca, I., Miceli, F., Franco, C., Canzoniero, L.M.T., et al. (2019). Epileptic Encephalopathy In A Patient With A Novel Variant In The Kv7.2 S2 Transmembrane Segment: Clinical, Genetic, and Functional Features. *Int J Mol Sci*. 20, pii: E3382. doi: 10.3390/ijms20143382.

Srivastava, S., Desai, S., Cohen, J., Smith-Hicks, C., Baranano, K., Fatemi, A., et al. (2018). Monogenic disorders that mimic the phenotype of Rett syndrome. *Neurogenetics*. 19, 41–47. doi:10.1007/s10048-017-0535-3.

Syrbe, S., Hedrich, U. B. S., Riesch, E., Djemie, T., Muller, S., Moller, R. S., et al. (2015). De novo loss- or gain-of-function mutations in KCNA2 cause epileptic encephalopathy. *Nat. Genet.* 47, 393–399. doi:10.1038/ng.3239.

Tabarki, B., AlMajhad, N., AlHashem, A., Shaheen, R., and Alkuraya, F. S. (2016). Homozygous KCNMA1 mutation as a cause of cerebellar atrophy, developmental delay and seizures. *Hum. Genet.* 135, 1295–1298. doi:10.1007/s00439-016-1726-y.

Torkamani, A., Bersell, K., Jorge, B. S., Bjork, R. L. J., Friedman, J. R., Bloss, C. S., et al. (2014). De novo KCNB1 mutations in epileptic encephalopathy. *Ann. Neurol.* 76, 529–540. doi:10.1002/ana.24263.

Trujillano, D., Bertoli-Avella, A. M., Kumar Kandaswamy, K., Weiss, M. E., Koster, J., Marais, A., et al. (2017). Clinical exome sequencing: results from 2819 samples reflecting 1000 families. *Eur. J. Hum. Genet.* 25, 176–182. doi:10.1038/ejhg.2016.146.

Vanderver, A., Simons, C., Schmidt, J. L., Pearl, P. L., Bloom, M., Lavenstein, B., et al. (2014). Identification of a novel de novo p.Phe932Ile KCNT1 mutation in a patient with leukoencephalopathy and severe epilepsy. *Pediatr. Neurol.* 50, 112–114. doi:10.1016/j.pediatrneurol.2013.06.024.

Weckhuysen, S., Ivanovic, V., Hendrickx, R., Van Coster, R., Hjalgrim, H., Moller, R. S., et al. (2013). Extending the KCNQ2 encephalopathy spectrum: clinical and neuroimaging findings in 17 patients. *Neurology*. 81, 1697–1703. doi:10.1212/01.wnl.0000435296.72400.a1.

Weckhuysen, S., Mandelstam, S., Suls, A., Audenaert, D., Deconinck, T., Claes, L. R. F., et al. (2012). KCNQ2 encephalopathy: emerging phenotype of a neonatal epileptic encephalopathy. *Ann. Neurol.* 71, 15–25. doi:10.1002/ana.22644.

Yamamoto, T., Imaizumi, T., Yamamoto-Shimojima, K., Lu, Y., Yanagishita, T., Shimada, S., et al. (2019). Genomic backgrounds of Japanese patients with undiagnosed neurodevelopmental disorders. *Brain Dev.* 41, 776–782. doi:10.1016/j.braindev.2019.05.007.

Yesil, G., Aralasmak, A., Akyuz, E., Icagasioglu, D., Uygur Sahin, T., and Bayram, Y. (2018). Expanding the Phenotype of Homozygous KCNMA1 Mutations; Dyskinesia, Epilepsy, Intellectual Disability, Cerebellar and Corticospinal Tract Atrophy. *Balkan Med. J.* 35, 336–339. doi:10.4274/balkanmedj.2017.0986.

Zhang, Q., Li, J., Zhao, Y., Bao, X., Wei, L., and Wang, J. (2017). Gene mutation analysis of 175 Chinese patients with early-onset epileptic encephalopathy. *Clin. Genet.* 91, 717–724. doi:10.1111/cge.12901.

Zhang, Y., Brown, M. R., Hyland, C., Chen, Y., Kronengold, J., Fleming, M. R., et al. (2012). Regulation of neuronal excitability by interaction of fragile X mental retardation protein with slack potassium channels. *J. Neurosci.* 32, 15318–15327. doi:10.1523/JNEUROSCI.2162-12.2012.

Zhang, Y., Kong, W., Gao, Y., Liu, X., Gao, K., Xie, H., et al. Gene Mutation Analysis in 253 Chinese Children with Unexplained Epilepsy and Intellectual/Developmental Disabilities. *PLoS One*. 10, e0141782. doi:10.1371/journal.pone.0141782.

Zhang, Z.B., Tian, M.Q., Gao, K., Jiang, Y.W., and Wu, Y. De novo KCNMA1 mutations in children with early-onset paroxysmal dyskinesia and developmental delay. Mov. Disord. 30, 1290–1292. doi: 10.1002/mds.26216.
