# Supplementary material for: Intellectual Disability and Potassium Channelopathies: A Systematic Review
Source: Front Genet. 2020 Jun 23;11:614. doi: 10.3389/fgene.2020.00614 (PMC7324798; doi:10.3389/fgene.2020.00614)
Supplement: Supplementary file 2 [file Presentation_1.pdf]

PRISMA Flow Chart

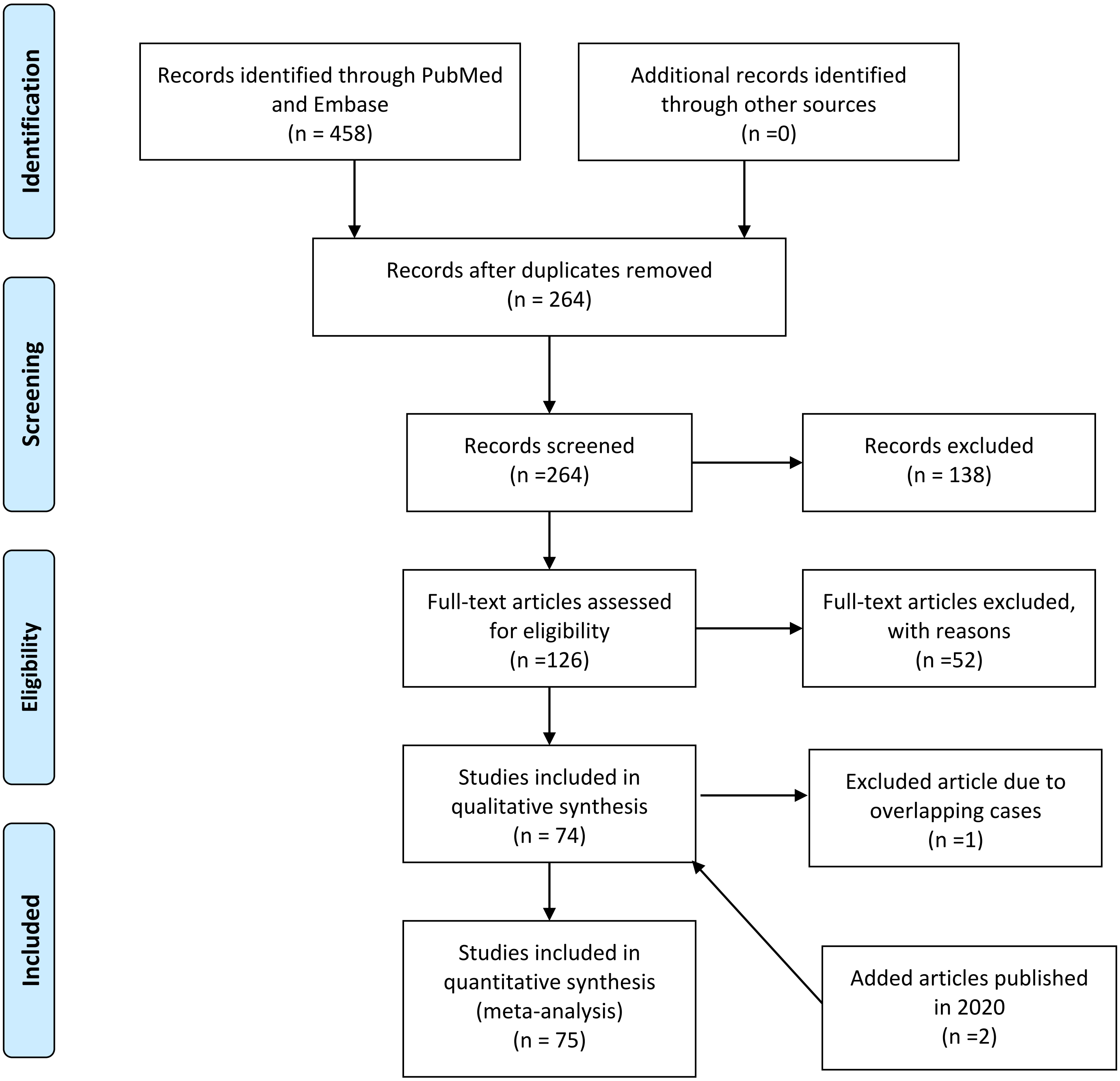

From: Moher D, Liberati A, Tetzlaff J, Altman DG, The PRISMA Group (2009). *P*referred *R*eporting *I*tems for *S*ystematic Reviews and *M*eta-*A*nalyses: The PRISMA Statement. PLoS Med 6(7): e1000097. doi:10.1371/journal.pmed1000097

For more information, visit www.prisma-statement.org.
