# Supplementary material for: Intellectual Disability and Potassium Channelopathies: A Systematic Review
Source: Front Genet. 2020 Jun 23;11:614. doi: 10.3389/fgene.2020.00614 (PMC7324798; doi:10.3389/fgene.2020.00614)
Supplement: Supplementary file 3 [file Data_Sheet_1.docx]

**Search strategies which were used.**

**PubMed**

1. MeSH Terms: potassium channels; intellectual disability.

Additional terms: intellectual; disability; intellectual disability; potassium channel; mental; potassium channels; channels; mental retardation; retardation; potassium; channel

1. MeSH Terms: potassium channels; intellectual disability.

Additional terms: potassium channels; intellectual disability; potassium; intellectual; channel; potassium channel; disability; channels.

1. MeSH Terms: potassium channels; internationality.

Additional terms: potassium channel; developmental; potassium channels; channel; globalize; globally; potassium; delay; delaying; globalization; global; globalized; globalism; delayed; globalizing; delays; developmentally; channels; globals; globalizes; internationality

**EMBASE**

1. ('pottasium channel' OR (pottasium AND channel)) AND ('intellectual disability'/exp OR 'intellectual disability' OR (intellectual AND ('disability'/exp OR disability))).
2. ('pottasium channel' OR (pottasium AND channel)) AND ('mental retardation'/exp OR 'mental retardation' OR (mental AND ('retardation'/exp OR retardation))).
3. ('pottasium channel' OR (pottasium AND channel)) AND ('global developmental delay'/exp OR 'global developmental delay' OR (global AND developmental AND delay)).
