# Supplementary figures and images for: Intellectual Disability and Potassium Channelopathies: A Systematic Review
Source: Front Genet. 2020 Jun 23;11:614. doi: 10.3389/fgene.2020.00614 (PMC7324798; doi:10.3389/fgene.2020.00614)

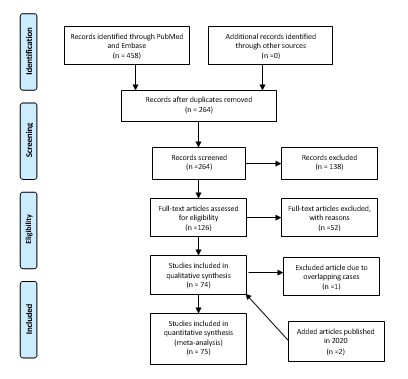

Supplement: Supplementary file 4 [file Image_1.jpg]
